# Learning to resist the urge: a double-blind, randomized controlled trial investigating alcohol-specific inhibition training in abstinent patients with alcohol use disorder

**DOI:** 10.1186/s13063-019-3505-2

**Published:** 2019-07-05

**Authors:** Raphaela M. Tschuemperlin, Maria Stein, Hallie M. Batschelet, Franz Moggi, Leila M. Soravia

**Affiliations:** 1 Translational Research Center, University Hospital of Psychiatry, University of Bern, Bern, Switzerland; 2 Center for Treatment of Addictive Disorders, Clinic Suedhang, Kirchlindach, Switzerland; 3 Department of Clinical Psychology and Psychotherapy, Institute of Psychology, University of Bern, Bern, Switzerland

**Keywords:** Alcohol use disorder, Alcohol-specific inhibition training, EEG, Cortisol, Go-NoGo Task (GNG), Stop-Signal Task (SST), Implicit Association Test (IAT), Residential treatment, Drinking outcome

## Abstract

**Background:**

Alcohol use disorder (AUD) leads to a significant individual and societal burden. To achieve higher therapy success rates, therapeutic interventions still need to be improved. Most current neuroscientific conceptualizations of AUD focus on the imbalance between an enhanced automatic reaction to alcohol cues and impaired inhibition. Complementary to traditional relapse prevention strategies, novel computerized training interventions aim to directly alter these processes. This study tests a computerized alcohol-specific inhibition training in a large clinical sample and investigates its effects on behavioral, experimental and neurophysiological outcomes. It also analyzes whether variations in inhibition difficulty and/or endogenous cortisol levels during training impact these effects.

**Methods:**

This is a double-blind, randomized controlled trial (RCT) with 246 inpatients with AUD participating. After baseline assessment, participants are randomly assigned to one of three computerized Go-NoGo-based inhibition training interventions (two alcohol-specific versions with different Go/NoGo ratios, or neutral control training) and one of two intervention times (morning/afternoon), resulting in six study arms. All patients perform six training sessions within 2 weeks. Endogenous cortisol is measured in 80 patients before and after the first training session. Inhibitory control and implicit associations towards alcohol are assessed pre and post training, at which point electroencephalography (EEG) is additionally measured in 60 patients. Patients’ alcohol consumption and relevant psychological constructs (e.g., craving, self-efficacy, treatment motivation) are measured at discharge and at 3-, 6- and 12-month follow-ups. Fifty healthy participants are assessed (20 with EEG) at one time point as a healthy control group.

**Discussion:**

This study investigates the effects of a computerized, alcohol-specific inhibition training for the first time in patients with AUD. Results should give insight into the effectiveness of this potential add-on to standard AUD treatment, including proximal and distal measures and effects on behavioral, experimental and neurophysiological measures. Information about working mechanisms and potential optimizations of this training are gathered through variations regarding difficulty of inhibition training and training time. This study may thus contribute to a deepened understanding of AUD and the improvement of its evidence-based treatment.

**Trial registration:**

ClinicalTrials.gov, ID: NCT02968537. Registered on 18 November 2016.

**Electronic supplementary material:**

The online version of this article (10.1186/s13063-019-3505-2) contains supplementary material, which is available to authorized users.

## Background

Alcohol use disorder (AUD) is a major public health problem which has a substantial impact on patients’ psychological, physiological and social functioning [1–4]. Severe AUD often remains a lifelong condition due to patients’ susceptibility to relapse [5–7]. Despite the development of valuable treatment programs [8, 9], one third of patients relapse within 1 year after treatment, highlighting the need for effective new interventions to be added to these programs.

Current models of substance use disorders (SUDs) are based on neuroscientific research into subcortical alterations (e.g., [10–12]) and psychological investigations on implicit processes [13], and postulate on an imbalance between subcortical, implicit processes and cortical control processes. Briefly, these models assume that automated (subcortical) cue-reactivity is enhanced, thus leading to a strong drive to consume, while the opponent (cortical) executive control is weakened, making it difficult to inhibit this impulse (e.g., [11, 14–16]). The neuroscientific findings that underlie these models include enhanced reactivity to substance-related cues in brain regions related to reward prediction and saliency processing (e.g., nucleus accumbens, amygdala), habits and motivation (dorsal striatum, orbitofrontal cortex) and alterations in brain regions related to inhibitory control (dorsolateral prefrontal cortex, inferior frontal cortex and anterior cingulate cortex) [17, 18]. There is an extensive body of literature indicating that SUD is associated with impaired performance in inhibitory control tasks [19–24]. Until recently, these tasks did not differentiate between inhibition in a substance-related versus neutral context. However, this distinction seems to be clinically relevant, given that the central malfunction leading to relapse is the inability to exert inhibitory control in a substance-related context. Recent studies have suggested that alcohol-related inhibition demands additional neuronal resources in patients with AUD and heavy drinkers [25–28].

To translate the findings of impaired inhibitory control and enhanced cue-reactivity into a therapeutic context, an inhibition training for eating disorders [29–31] has been adapted into a novel alcohol-specific inhibition training and introduced into the field of AUD research [32, 33]. During this short, computerized, behavioral training, alcohol-related stimuli are consistently paired with a stopping response. Such an intervention might thus improve inhibitory control by training it on an explicit as well as a habitual level and possibly reduce cue-reactivity through stimulus devaluation.

The effect of one or two of these training sessions on alcohol consumption has been tested in regular drinkers [34, 35] and non-clinical, heavy drinkers [32, 33, 36, 37]. When alcohol consumption was assessed immediately after the training session, two studies found decreased consumption [35, 36], one study observed a non-significant trend in that direction [33], another study reported longer latency until participants took the first sip [37], and one study found no effects [34]. Of those studies assessing alcohol consumption 1 or 2 weeks post intervention, three studies reported decreased consumption after alcohol-specific inhibition training [32, 33, 37]. Note, however, that in two of these studies, the intervention was compared to a control condition, which could potentially have fostered alcohol consumption within the control group [32, 33]. Two other studies found no significant effects [34, 35]. In summary, while the effects of this training are inconsistent in non-clinical samples, it might be more effective in clinical populations where (1) the motivation to change drinking behavior is higher and (2) the impairment targeted by the training is assumed to be more pronounced at baseline. Therefore, it is important to test the feasibility and efficacy of the training in clinical populations with AUD. The present randomized controlled, double-blind study aims to close this gap, and includes follow-up measurements of drinking behavior and related concepts up to 12 months after treatment discharge, allowing assessment of potential clinically relevant long-term effects.

There are two putative working mechanisms by which alcohol-specific inhibition training is thought to be effective. The first is through enhancing inhibitory capacities in the context of alcohol-related cues, which can be assessed with inhibitory tasks, such as the Go-NoGo Task (GNG) or the Stop-Signal Task (SST). The second putative working mechanism regards devaluation of the stimuli. It is based on the *Behavior Stimulus Interaction Theory* [38], which states that the repeated stopping response to the rewarding (alcohol, in this context) stimuli causes a devaluation thereof [31, 33]. This mechanism is thus thought to alter the automatically attributed appeal of alcohol-related stimuli, an effect which can be measured with the Implicit Association Test (IAT).

With respect to the devaluation mechanism, the first two studies were consistent with predictions of the *Behavior Stimulus Interaction Theory* in their reports that participants’ implicit attitudes shifted towards a more negative evaluation of alcohol-related stimuli after alcohol-specific inhibition training [32, 33]. However, this finding could not be replicated in the four subsequent studies [34–37].

In regard to an inhibition-related working meachanism, those studies using the respective measures found no effects of GNG-based inhibition training on an SST [32, 37] nor flanker task [34]. However, these tasks measure slightly different inhibitory aspects (e.g., action cancellation in case of the SST) than the one affected during the GNG-based training (action restraint). The only study which assessed inhibitory functioning in the same form as targeted during training (i.e., measurement of inhibitory control with a GNG after a GNG-based training) reported no effects on reaction times during Go trials. Unfortunately, data on errors of commission (EOCs), which are a commonly used indicator of inhibitory capacities and an essential and proximal experimental outcome of a GNG-based inhibition training, were not reported in this study [36] and are thus still missing. Another important aspect concerns Go/NoGo ratio incorporated during training sessions. With one exception [34], most studies emloyed equiprobable GNG tasks with a Go/NoGo ratio of 50/50 during the training session. Such a ratio is unusually balanced for GNG tasks, where NoGo responses usually only make up 10–20% of the trials (e.g., [23, 24, 39]) in order to establish a high tendency to respond while making inihibition more demanding. Thus, it is possible that these versions failed to create the context of a prepotent Go response, in which inhibition could effectively be trained. The present study aims to extend prior research on potential working mechanisms by comparing two versions of this alcohol-specific inhibition training differing solely in the Go/NoGo ratio (thus in inhibition difficulty) and by comparing these versions’ effects on inhibitory capacities as well as on implicit associations.

With regard to other clinically important psychological variables, research has shown that outcome expectancies [40], self-efficacy to remain abstinent [41], motivation to change alcohol use [42] and craving [43] predict drinking outcomes at 1-year follow-up. These variables might thus act as potential mediators of effects in a clinical study. Furthermore, if the alcohol-specific inhibition training influences implicit associations towards alcohol, it might also affect explicit expectancies and/or subjective craving. The present study will, therefore, include these variables as potential outcomes and mediators in explorative analyses.

Taken together, the present study investigates the effects of an alcohol-specific inhibition training for the first time in a clinical sample using a randomized controlled, double-blind design with long follow-up periods. In doing so, the study contributes to research on potential working mechanisms by comparing two versions of the training which differ only in the Go/NoGo ratio and by the comparison of their effects on inhibitory functions and implicit associations. Explorative analyses will target psychological variables, such as craving, outcome expectancies, self-efficacy and motivation to change alcohol use. As the training’s rationale is anchored in neuroscientific research, we will also monitor neurophysiological effects with multi-channel electroencephalography (EEG) in order to investigate whether the training changes the neurophysiological correlates of alcohol-specific inhibition and/or implicit associations. Finally, prior research has shown that endogenous cortisol is a consolidation enhancer [44], which peaks in the morning [44, 45] and has been shown to improve effects of other learning-based therapies [46]. Since training procedures rely on learning processes affected by endogenous cortisol, we are, therefore, interested in whether its levels influence training effects.

### Study aim

In the present project, the *In*hibition *Tra*ining (INTRA) study, 246 recently abstinent patients with AUD attending an inpatient treatment program will be randomly assigned to one of two alcohol-specific inhibition training groups (each with a different Go/NoGo ratio) or to a control group. Our aim is to examine whether variations of inhibition training have a positive effect on drinking behavior, implicit attitudes, and neurophysiological reactivity to alcohol-related stimuli. Thus, a subgroup of patients will additionally undergo EEG recording before and after the intervention so that neurophysiological effects of the training can be assessed and related to clinical outcomes. In addition, 50 healthy controls (with EEG measurement of 20) will be assessed once to compare patients’ pre-training data. Since training effects rely on learning processes, the influence of endogenous cortisol level (a consolidation enhancer, which peaks in the morning and decreases in the course of the day [44]) on training outcome will be investigated by varying the time of day in which the training is performed. All patients’ inhibition and implicit associations towards alcohol will be measured immediately before and after the training. We will also measure the training’s effects on proximal outcome variables (e.g., implicit associations, inhibitory control, abstinence-related self-efficacy, craving) post training, and distal outcome variables (e.g., percentage of days abstinent – PDA; heavy-drinking days – HDD; and time to first drink – TFD) at 3-, 6- and 12-month follow-ups.

For the first time, this trial will investigate the therapeutic potential of an alcohol-specific inhibition training as a therapeutic supplement in a sample with patients suffering from severe AUD. In doing so, the impact on training efficacy of time of training and Go/NoGo ratio will additionally be investigated and the underlying neurophysiological mechanisms of the training elucidated. Moreover, potential AUD-related psychological constructs will be explored. The project thus aims to contribute to the improvement of the evidence-based treatment of AUD.

### Research questions

Based on prior research, the following five research questions are examined:

1. Does the alcohol-specific inhibition training (Alc-IT, compared to the control intervention) reduce PDA and HDD and/or extend the time to first drink (TFD) at 3-months’ follow-up (primary outcomes)?
2. What is the effect of the Alc-IT on behavioral experimental parameters?
  - Does Alc-IT decrease positive implicit associations compared to the control training?
  - Does Alc-IT enhance response inhibition compared to the control training?
3. Are the neurophysiological correlates of alcohol-specific inhibition and implicit associations towards alcohol changed by Alc-IT?
4. Does endogenous cortisol moderate the effect of the Alc-IT?
5. In addition, explorative questions investigate whether the effects of the training on outcomes are mediated by AUD-related psychological constructs, such as alcohol-related self-efficacy, craving or motivation

## Methods

### Study design and setting

**Fig. 1:**
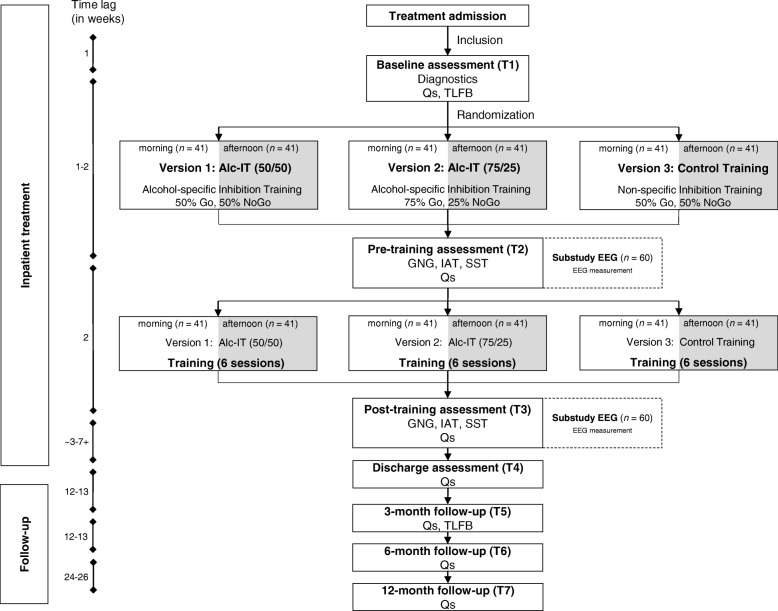
Study design of the INTRA study. Note that for the cortisol substudy, salvia-cortisol samples will be collected before and after the first training session. *Alc-IT* alcohol-inhibition training, *EEG* electroencephalography, GNG *Go-NoGo Task*, *IAT* Implicit Association Test, *INTRA* Abbreviation of the study name: INhibition TRaining in Alcohol use disorder, *n* sample size, *SST* Stop-Signal Task, *TLFB* Timeline-follow-back, *Q* questionnaire

The present study is a multicenter, double-blind, randomized controlled trial (RCT) with six conditions. The interventions consist of two alcohol-specific inhibition trainings (with different Go/NoGo ratios) and one control inhibition training that is not context specific. In each version of the training, six sessions will be performed over the space of 2 weeks. Next to the three versions of the training, different samples will perform the training in different times of the day (morning/afternoon), resulting in a total of six conditions. Between the three study sites, 246 abstinent inpatients with AUD will be recruited. To compare our patient sample to the healthy population, we will measure 30 (behavioral) and 20 (EEG) healthy participants at one point in time. The duration of the study from the first inclusion to the last participant’s follow-up is 36–40 months. Recruitment, randomization, and baseline measurements (T1) will take place at one of the three study sites at the beginning of the regular inpatient program. All patients will have undergone detoxification prior to entering this inpatient program. Pre-training assessment (T2) is usually performed by the end of the second week or at the beginning of the third week of the inpatient stay and is followed by the 2 weeks, during which the six training sessions of the intervention or the control training are performed. Post-training assessment (T3) takes place 1 to 4 days after the last training session. At discharge (T4), the last measurement during inpatient program is performed. The duration of the inpatient stay typically varies between 8 and 12 weeks, depending on individual and institutional factors. Follow-up assessments take place 3 (T5), 6 (T6) and 12 (T7) months after discharge and consist of a questionnaire battery and a telephone interview (see also “Procedure” and Fig. 1).

### Participants

#### Patients

We will screen all patients who previously completed a detoxification and then entered an alcohol-specific inpatient treatment program at one of three specialized addiction treatment centers in Switzerland, namely the Suedhang Hospital (Bern); the Forel Hospital (Zuerich) and the Psychiatric Hospital of Muensingen (Bern). Inclusion criteria are age 18–60 years and abstinence from alcohol for at least 4 weeks prior to the first training session. Exclusion criteria are other main psychiatric diagnoses than AUD (comorbidities are allowed as long as AUD is considered to be the main diagnosis), other severe SUD (except nicotine; Drug Use Identification Test (DUDIT [47, 48]) ≥ 25 per substance), no diagnosed neurocognitive problems (e.g., Korsakoff syndrome) in the medical history, current medical conditions preventing participation (e.g., acute infectious diseases), the inability to read and understand the participant’s information and the enrollment of an investigator, their family members, employees and other dependent persons. Written informed consent will be obtained from all patients at the beginning of the study.

#### Healthy controls

In order to compare behavioral data of the patient sample to that of a healthy population, 50 healthy controls will be measured at one point of time (20 with EEG). Inclusion criteria for healthy controls are age 18–60 years, non-problematic drinking behavior (Alcohol Use Disorders Identification Test (AUDIT [49]) < 8; Alcohol Use Disorder Scale (AUD-S [50]) < 2)), and no signs of psychopathology (Brief Symptom Check List (BSCL [51, 52],) GSI_t-value_ ≤ 63). Exclusion criteria consist of current treatment for a psychiatric diagnosis and/or psychopharmacological medication, treatment for SUD in the past, problematic substance use (except nicotine; DUDIT ≥8 per substance, e.g., cannabis), neurocognitive problems, poor health conditions, the inability to read and understand the study information. In the substudy EEG, additional exclusion criteria are the occurrence of AUD in first-degree relatives and hearing impairments. Written informed consent will be obtained from all healthy controls prior to inclusion. For a tabular overview of all in- and exclusion criteria, see Additional file 1.

### Stimulus material

#### Stimuli for training, GNG and IAT

A total of 40 pictures of alcoholic beverages, water and neutral objects were created by the study team. All pictures were photographed with a high-resolution camera (Nikon D810, Nikon Inc., Tokyo, Japan) in a neutral setting with a white background and the same light conditions (two spotlights). Water pictures are the same for all participants, whereas alcohol pictures will be matched to the individual’s drink of choice. For this purpose, three sets with either beer (seven lager, one wheat), wine (two red, two white, two rosé, two sparkling wines) or spirits (two vodka, two whiskey, two liquor, one gin, one tequila) were generated. Each of the four beverage sets includes eight pictures of brands commonly consumed in Switzerland, with all pictures comprising a bottle or can and a full beverage-specific glass (for examples, see Additional file 2).

The eight neutral stimuli are everyday objects (e.g., a flashlight plus battery) and were selected by complexity, shape and familiarity. All pictures were edited with GIMP software (GIMP 2.8.18, retrieved from https://www.gimp.org) and adjusted for size, centralization, brightness and background to ensure picture similarity.

#### Stimuli for SST

For the third task, stimulus material consist of 16 pictures (344 × 400 pixel) of a database and are characterized by their alcohol relatedness, valence, craving, arousal, luminance, colors and visual complexity [53]. Four pictures of each drink of choice (beer, wine and spirits), shot in (social) daily situations within a building, and four pictures of everyday objects (e.g., a hydrant) located outdoors, were selected. This additional stimulus material was chosen to test for generalization of the effect of our intervention to a novel inhibition task and to new stimuli.

### Intervention

Participants in both versions of the Alc-inhibition training (Alc-IT) are, as the name suggests, trained in alcohol-specific inhibition: pictures of alcoholic beverages will be consistently paired with a NoGo cue, while Go cues will be distributed among the other picture types (water, neutral). In contrast, for patients in the control training group, all three picture types will be paired equally often with NoGo cues and Go cues (see Table 1).

- *Alc-IT (50/50)*: the first training group will operate with a Go/NoGo ratio of 50/50, as implemented in prior studies [32, 33]: This original version of the Alc-IT will include 80 alcoholic NoGo trials as well as 80 non-alcoholic Go trials. In order to match both Alc-IT versions in training length while keeping the number of Alc-NoGo pairings constant, an additional 80 neutral Go trials and 80 neutral NoGo trials were included
- *Alc-IT (75/25)*: the second version of the Alc-IT will operate with a Go/NoGo ratio of 75/25. It will equally include 80 alcoholic NoGo trials and 80 non-alcoholic Go trials, but contrary to Alc-IT (50/50) 160 neutral Go trials will complete the set. Both Alc-IT versions thus include the same number of Alc-NoGo pairings and are of the same length, but differ in their Go/NoGo ratio and thus in the difficulty regarding inhibition control
- *Control training*: the control group will receive a training that consists of 80 non-alcoholic, 80 alcoholic, and 160 neutral trials. This control training is thus a non-specific inhibition training which is of equal length to other two groups and includes the same number of alcohol-related pictures in it, but all pictures will appear with equal probabilities as Go or NoGo trials while the Go/NoGo ratio is kept at 50/50

**Table 1:** Distribution of trial types in the three training versions

	Alc-IT (50/50)		Alc-IT (75/25)		Control training	
	Trial type		Trial type		Trial type	
Picture types	Go	NoGo	Go	NoGo	Go	NoGo
Alcohol	–	80	–	80	40	40
Water	80	–	80	–	40	40
Neutral	80	80	160	–	80	80
Total trials	320		320		320	

All three versions of the inhibition training were programmed with Inquisit 5 (Version 5.0.5.0., Millisecond Software, Inc., Seattle, WA, USA). They are based on the script by Houben, Havermans [32], Houben, Nederkoorn [33], which was gratefully received by the authors and adapted to their own version. Go and NoGo cues are represented by the letter “p” or “f” next to one of the four corners of the alcohol, water or neutral pictures (1047 × 1080 pixel), whereby the assignment to the letters will be counterbalanced across participants. Participants will be instructed to press the space bar when a Go cue has been displayed and to refrain from pressing it when a NoGo cue has appeared. Each version of the training lasts approximately 13 min and consists of 320 trials, which are presented in a randomized order. A trial includes the simultaneous presentation of a picture and a cue (paired in accordance with the intervention version) and is visible on screen for 1500 ms. Participants are instructed to respond as fast as possible, unless the picture is paired with a NoGo cue. 500 ms after the (non)response visual feedback will be given, with a green circle indicating a correct (non)response, and a red cross indicating an incorrect (non)response. Based on data by Eberl et al. [54], which compared learning outcome after varying numbers of sessions of computerized training in AUD patients, participants will perform six training sessions.

### Interviews and questionnaires

#### Demographics

Relevant demographics (e.g., age, gender, education) as well as relevant information about past AUD treatment or other mental health problems will be assessed.

#### Diagnostics

The German version of the semi-structured interview of the *Diagnostic Expert System for Psychiatric Disorders* (DIA-X, adapted to the *Diagnostic and Statistical Manual of Mental Disorders* (DSM-5); [55])) specifically for AUD will be performed by a trained member of the study team. The original DSM-IV version was modified to meet the DSM-5 criteria, whereby the symptom craving was added and the question about legal issues was removed.

#### Alcohol use disorder and other substance use

The screening questionnaire *Alcohol Use Disorders Identification Tests* (AUDIT; [49]) from the World Health Organization (WHO) consists of 10 items on recent alcohol use, AUD symptoms, and alcohol-related problems.

With the *Alcohol Use Disorder - Scale* (AUD-S; [50]; adapted to DSM-5), self-rated AUD symptoms are measured, whereas this adapted version additionally includes craving as a criteria.

To assess patients’ alcohol use in the last 90 days prior to their current detoxification, an adapted version of the *Health and Daily Living Form* (HDL; [56]) will be used, which was also employed in a large, Swiss multicenter study [57, 58]. Patients will be asked about their consumption frequency and quantity of beer, wine, liquor and spirits, resulting in total number of standard drinks (SD; 1 SD equates to 3 dl beer, 1 dl wine, 2 cl spirits/liquor, or 10 g of pure alcohol) consumed per day. Separate items assess the PDA and the number of HDD for the last 90 days.

To measure the drinking behavior for each of the past 90 days, the *Timeline Follow-back* (TLFB; [59, 60]) method will be performed, which will be administered by an interview (face-to-face at T1 or telephone at T5) with a trained member of the study team. As in the HDL, the amount of alcohol per day will be assessed in standard drinks per day, but in contrast is conducted through a guided, standardized interview. The TLFB provides a report of drinking pattern, including PDA, HDD and TFD.

With the *Drug Use Identification Test* (DUDIT; [47, 48]), participants will be screened for substances (except nicotine) other than alcohol. Patients with other severe substance use and healthy controls with a problematic substance use are not eligible for this trial (see also “Inclusion/exclusion” criteria).

#### Alcohol-related constructs

Alcohol craving will be measured by the *Obsessive Compulsive Drinking Scale,* (OCDS-G; [61]). The items of the OCDS-G are divided into two subscales: control and consequences, and drinking obsessions.

As a second measure for craving, three specific questions will be asked [57, 58], which are scored from 0 (non-existent or never) to 10 (very strongly or always): “How strong was your urge to drink alcohol (on average) during the past 7 days?”; “Think of the moment during the past 7 days, in which the urge to consume alcohol was the highest. How strong was the urge?”; and “How often did you have the urge to drink alcohol during the past 7 days?”

Motivation to change alcohol use will be assessed with the *Stages of Change Readiness and Treatment Eagerness Scale* (SOCRATES; [62]), whereby only the subscale *Taking Steps* will be used. It indexes whether the person has already taken action to positively change their drinking behavior.

A second measure for motivation consists of three items on an 11-point Likert scale, summed up as *Motivation* [41, 63, 64]. Inspired by the framework of motivational interviewing [64], the first question “How important is it for you not to drink alcohol? What do you think about that at this very moment?” [63] will be followed by the statement “I will do everything to get my alcohol problem under control.” [64]. Third, general self-efficacy will be assessed with one question: “How confident are you that you will be completely abstinent in one year from now?” [41].

A broader concept of alcohol-specific self-efficacy will be measured with the *Alcohol Abstinence Self-Efficacy Scale* (AASE-G [65]), which assesses the patients’ expectations concerning their ability to remain abstinent from alcohol in specific high-risk situations.

Positive and negative alcohol outcome expectancies are quantified with the *Comprehensive Alcohol Expectancy Scale* (CAEQ [66, 67]). A reduced version [67] will be used to assess five dimensions of alcohol expectancies: social assertiveness and positive affect; tension reduction; cognitive impairment and physical discomfort; aggression; and sexual enhancement.

As has been done before [57, 58], *Drinking Goals* will be assessed by asking the patients whether their personal drinking goal is (1) total abstinence and hence no future alcohol use, (2) abstinence with occasional lapses, or (3) controlled and responsible drinking.

Finally, with a self-created *Discharge Questionnaire,* all patients will be asked about occurring relapses during their treatment. Participants will be informed that this information is confidential and will not be shared with the staff of the relevant hospital.

#### Psychopathology

General psychopathology will be assessed with the *Brief Symptom Check List,* (BSCL, formerly BSI; [51, 52]), which comprises of nine primary symptom dimensions (e.g., depression, anxiety, hostility) and three global indices (e.g., global severity index).

Apart from this general measure, several screenings for specific psychopathological syndromes which are often comorbid with AUD will be implemented: the intensity of anxiety symptoms will be assessed with the *Beck Anxiety Inventory* (BAI [68, 69]), and for qualification of the severity of depressive symptoms, the *Beck Depression Inventory* (BDI-II [70, 71]) will be administered. The *ADHD Self Report Scale* (ASRS-V1.1 [72, 73]) assesses symptoms of Attention Deficit and Hyperactivity Disorder (ADHD). The *PTSD Screening Scale* (PSS; [74, 75]) qualifies symptoms of posttraumatic stress disorder (PTSD).

Additionally, stress (due to loss or experience of negative events) and coping will be assessed with the *Stress and Coping Inventory* (SCI [76]). Subjectively perceived quality of life will be measured with three subscales (global scale, physical and psychological health) from the short version of the *World Health Organization Quality of Life* questionnaire (WHOQOL-BREF [77, 78]).

#### Traits

Relevant traits linked to AUD are of special interest: Impulsivity will be measured with the short questionnaire *Scale for Impulsive Behavior* (I-8 [79]). Sensation seeking will be qualified with the *Need Inventory of Sensation Seeking* (NISS [80])*,* which includes two subscales (need for stimulation and avoidance of rest). To assess *Antisocial Personality Disorder* (ASPD), an adapted questionnaire from the Mini-International Neuropsychiatric Interview (M.I.N.I. [81], also employed in the C-Surf cohort study [82]) will be used. To quantify motivational systems (activation/inhibition), underlying behavior and affect, the *Behavioral Inhibition System/Behavioral Approach System Scales* (BIS/BAS [83]) will be used as in the C-Surf cohort study [84] in a version adapted from the validated German version [85].

#### Other

Additionally, two other measures will be used: experienced emotions as well as the capability of the participant to experience and regulate these emotions will be measured with the *Emo-Check* [86, 87]. The *Trail Making Test A and B* (TMT A & B [88]) screens for neuropsychological executive function by assessing visual attention and task switching. Previous research with preclinical samples indicates that executive functions could influence the relationship between alcohol-related implicit cognitions and addictive behaviors ([89, 90], but see [54]). Consequently, the TMT will be used as a covariate in the statistical analyses.

### Experimental tasks

Both the computerized experimental tasks and response logging are programmed and administered with E-Prime 2.0 (EP2Pro2.0.10.356, Psychology Software Tools, Sharpsburg, PA, USA). With the picture size (344 × 400 pixel) being equal for all three tasks, the set of alcohol-related pictures will be matched to the individual’s drink of choice (beer, wine, or spirits).

#### Go-NoGo Task (GNG)

This task measures response inhibition in both an alcohol-specific and a neutral context. In the modified version of Stein et al. [25], eight pictures of alcoholic beverages (beer, wine, or spirits, according to the participants drink of choice) and eight neutral beverages (water) are presented on screen at a presentation rate of 1 Hz. The pictures are displayed on-screen for 900 ms and the inter-stimulus interval is 100 ms. Participants are instructed to click a button as soon as a stimulus appears on screen (Go trial) unless the same stimulus appears twice in a row (NoGo trial). Each of the 16 pictures will be presented 60 times during the task: 52 times as a Go trial and eight times as a NoGo trial, resulting in a Go/NoGo ratio of 6.5. This ratio is intended to establish a high tendency to respond, making action restraint difficult. In total, the whole experiment thus includes 960 trials (416 neutral Go trials, 416 alcohol-related Go trials, 64 neutral NoGo trials, and 64 alcohol-related NoGo trials), which are presented in a pseudorandomized order, controlling for position, sequential order, and requiring a minimum of two Go trials between two NoGo trials. Half way through, participants are allowed a pause of a self-determined length. The whole task takes approximately 18 min to complete.

#### Stop-Signal Task (SST)

As a second measure of response inhibition, a Stop-Signal Task (SST) will be performed, which includes four pictures of alcoholic beverages located inside a building and four neutral pictures of objects located outside. After a fixation cross, which lasts 460 ms, the individual pictures will appear on a computer screen and participants will be instructed to press the button “a” (left) or “l” (right) as soon as possible to indicate whether the picture was taken inside (confounded with alcohol-related content) or outside a building (confounded with neutral content). During instructions, this pairing of location (inside, outside) and content (alcohol, neutral) is not mentioned. An exception to the rule is to be made when an auditory stop signal occurs (NoGo; 25% of the trials). This stop signal starts with a delay of 100, 200, or 300 ms (each comprising one third of NoGo trials) after stimulus onset. As this stop signal appears after the picture, and not simultaneously with it, as during the GNG or the training, task performance in the SST depends on action cancellation and thus measures a slightly different aspect of response inhibition. After completion of two practice blocks each, two test blocks comprising 240 trials each (180 Go and 60 NoGo trials) will be completed. The key allocation is counterbalanced, and the order of trials is pseudorandomized. As an indicator of response inhibition, the stop-signal reaction time (SSRT) will be computed for alcohol-related and non-alcoholic pictures according to the algorithm described in Houben, Havermans [32].

#### Implicit Association Test (IAT)

Implicit associations between alcohol and affective attributes will be measured with an alcohol-specific IAT [91]. In a *valence IAT*, pictures of alcoholic beverages are either paired with a positive (e.g., funny) or a negative (e.g., dull) word. Participants must classify if a stimulus either belongs to one of the target categories (pictures of alcoholic drinks vs. waters) or to one of the affective categories (positive vs. negative verbs) with response buttons on a keyboard (left “a” vs. right “l”). The eight alcoholic and eight water pictures are the same as in the inhibition training (see “Stimulus material”). In accordance with previous preclinical studies [32, 33], the affective categories consist of eight positive (happy, jolly, energetic, funny, sociable, attractive, cheerful, smart) and eight negative (dull, miserable, sick, depressed, unhappy, disgusting, angry, foolish) attributes. The German translation (glücklich, ausgelassen, dynamisch, lustig, gesellig, attraktiv, fröhlich, klug, lustlos, miserabel, übel, deprimiert, unglücklich, widerlich, wütend, dumm) were validated by three independent native-English speakers. During “alcohol-positive” blocks, stimuli from the categories alcohol and positive are represented by one response button, while water and negative attributes require pressing the other response button. Contrary to this, during “alcohol-negative” blocks, stimuli from the alcohol and negative category share one response button, while water and positive attributes are indicated with the other response button. Based on the rationale that associated concepts (e.g., alcohol and positive attributes) are more easily combined than unrelated concepts (e.g., alcohol and negative attributes), reaction time differences between these two blocks can be used to assess the association strength between target and affective categories. According to the developers [91], a significant difference in reaction time between both examples would indicate strong positive associations towards alcohol.

For this study, task development in E-Prime was based on an adapted version used by Egenolf et al. [92]. To control for sequence effects of the starting target concepts (alcohol, water) both IAT versions consist of 14 blocks. All blocks are interposed with rest periods of a self-determined length. Initially, and whenever key assignment changes, participants will undergo two or three practice blocks consisting of 16 trials each: First, they classify the target concepts (alcohol, water) and/or the affective categories (positive, negative) to left (“a”) and right (“l”). Second, they practice the combination of target concepts and affective categories. After these blocks, a longer test block of 64 trials follows (for an overview of the procedure, see Additional file 3). To remind participants of the current mapping rule, category labels will be presented in the upper two corners of the screen. Stimuli will appear on-screen for maximum 1750 ms or until an answer is given, followed by feedback (for 200 ms). The interstimulus interval will be 250 ms. The IAT consists of 416 trials and lasts 10–20 min depending on the individual’s speed and duration of rest periods. Reaction time analyses will be conducted according to the improved scoring algorithm by Greenwald, Nosek et al. [93].

### Procedure patients

**Fig. 2:**
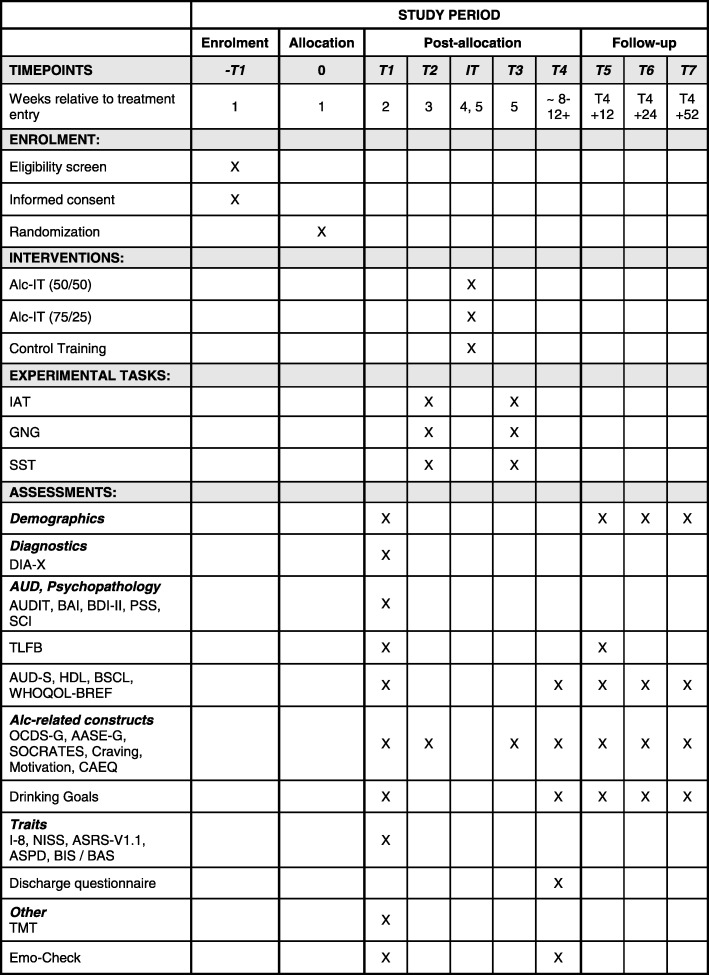
Enrollment, interventions and assessments at each time point for patients in the INTRA study. A schematic outline of the enrollment, interventions and assessments that each participant of the main INTRA study will undergo. *AASE-G* Alcohol Abstinence Self-efficacy Scale – German version, *Alc-IT* alcohol-inhibition training, *ASPD* antisocial personality disorder, *ASRS-V1.1* Adult ADHD Self-Report Scale, *AUDIT* Alcohol Use Disorders Identification Test, *AUD-S* Alcohol Use Disorders Scale, *BAI* Beck Anxiety Inventory, *BDI-ll* Beck’s Depression Inventory, *BIS/BAS* Behavioral Inhibition System/Behavioral Approach System Scale, *BSCL* Brief Symptom Check List, *CAEQ* Comprehensive Alcohol Expectancy Questionnaire; Craving (Likert scales), *DIA-X* Diagnostic Expert System for Psychiatric Disorders, *Emo-Check* Assessment of Emotion and Emotion regulation, *GNG* Go-NoGo Task, *HDL* Health and Daily Living Form, *IAT* Implicit Association Test, *IT* inhibition training, *I-8* Scale for Impulsive Behavior, Motivation (Likert scales), *NISS* Need Inventory of Sensation Seeking, *OCDS-G* Obsessive Compulsive Drinking Scale – German version, *PSS* PTSD Symptom Scale, *SCI* Stress and Coping Inventory, *SOCRATES* Stages of Change Readiness and Treatment Eagerness Scale, *SST* Stop-Signal Task, *TLFB* Timeline Follow-back, *TMT* Trail Making Test A and B*, WHOQOL-BREF WHO Quality of Life Scale (brief version), −T1* before treatment admission, *T1* baseline, *T2* pre-training, T3 post training, *T4* discharge, *T5* 3-month follow-up, *T6* 6-month follow-up, *T7* 12-month follow-up

The study duration from the admission of the first participant to the last participant’s follow-up is 36–40 months. The duration of participation for an individual study participant is commonly 14–16 months depending on the duration of the inpatient stay. Apart from the main study, the substudies *cortisol* and *EEG* are integrated in the main study procedure. During the standard inpatient treatment, the assessments T1 to T4 will take place. After discharge, and at each of the three follow-up time points, questionnaires will be sent to each participant, and they are additionally contacted by telephone (see also Fig. 1). For an overview about the single time points, consult the Standard Protocol Items: Recommendations for International Trials (SPIRIT) Figure (Fig. 2). The SPIRIT Checklist is also provided as Additional file 4.

#### Recruitment

At treatment admission, all patients’ medical records will be screened for eligibility for participation in the study. If all inclusion criteria and no apparent exclusion criteria are fulfilled, the patient will be invited to an information appointment during the first treatment week. Therein, the study will be explained, questions will be answered, and lastly, the person is asked if they would participate. For those having given informed consent, we will screen for other severe substance use disorders using the DUDIT [47].

#### T1: Inclusion and baseline assessment

During the second treatment week, a member of the study team will conduct an interview to confirm the AUD diagnosis (DIA-X adapted to DSM-5 criteria; [55]). To assess the drinking quantity of the 90 days prior to detoxification, the TLFB interview will be conducted [59, 60]. Furthermore, a psychological assessment with a test battery consisting of questionnaires will also take place at this time point.

#### Randomization and blinding

Patients will be randomly assigned to one of the three study arms, with the aim of generating equally sized groups. Block randomization with variable block sizes will be stratified according to gender and age (age groups: 18–25, 26–35, 36–45, 46–55 and 56–60 years). The stratified randomization list will be generated with Matlab (Version 2017a, MathWorks, Inc., Natick, MA, USA), and stored in one document. The participants’ allocation to a study arm will be done by an independent investigator who will administrate and conceal the list. Thus participants, investigators, care providers and members of the study team will be blind to the allocation schedule.

#### T2: Pre-training assessment

Prior to the training, all subjects will undergo an assessment including a series of questionnaires as well as experimental tests measuring implicit associations towards alcohol (IAT) and inhibitory control (GNG, SST). This will take place at the end of the third treatment week. A total of 60 patients (for the EEG substudy) will additionally be assessed with multi-channel EEG while completing the experimental tasks. As the intense confrontation with alcohol pictures may lead to tension and induce craving in patients with AUD, we will monitor our participants’ stress and craving on an 11-point Likert scale (0 = not at all, 10 = very strong) immediately before and after the assessment. In the case of significant stress or craving levels, the medical staff on duty will be contacted and informed.

#### Training (intervention)

During weeks 4 and 5 of the treatment, all patients will undergo six short training sessions according to their allocated training version (Alc-IT (75/25), Alc-IT (50/50) or control group, either in the morning or afternoon). All training sessions will be distributed over 2 weeks, performed in a group setting, and monitored by a member of the study team. At the beginning of the first session, participants undergo a short practice version to confirm that they understood the task. Before and after each training session, stress and craving will be assessed (see also T2). At the end of each training session, the study member will take note of the participants’ reaction time and error rate, with the aim to maintain motivation. In order to assess saliva cortisol concentration for the cortisol substudy, saliva samples of 80 patients of the Suedhang Hospital will be collected before and after the first training.

#### T3: Post-training assessment

One to 4 days after the last training session, patients will undergo the same assessment as in T2 (including questionnaires and experimental tasks). The 60 participants of the EEG substudy again participate in the EEG recording during the experimental tasks. Before and after the measurement, self-reported stress and craving will be assessed (see also T2).

#### Discharge assessment (T4)

Upon discharge from inpatient treatment, psychological parameters are measured again with the questionnaires used at previous time points. At the end of T4, the procedure for the follow-up measurements will be explained.

#### 3-month follow-up (T5)

Three months after discharge, all participants will be contacted by telephone. The primary outcome variables will be assessed in a short telephone interview, and an appointment for the TLFB interview will be arranged if it cannot be carried out at the time of the call. Parallel to this telephone interview, a questionnaire battery will be sent to the patients’ private address.

#### 6- and 12-month follow-up (T6, T7)

Six (T6) and 12 (T7) months after discharge, a similar procedure as in T5 will be performed. At both time points, participants will receive the questionnaire battery by post and will be called by telephone to assess the most significant outcome variables.

#### Procedure to enhance response rates at the follow-up assessments (T5, T6, T7)

To achieve high response rates, a meticulous plan will be pursued for each follow-up assessment: Participants will be contacted by telephone starting 10 days prior to the target day (90, 180 and 365 days after discharge) to announce the upcoming assessment. Should three contact attempts fail, a text message is sent. In case of no response, they are called another seven times, and a set of questionnaires will be sent shortly before the target day. If these questionnaires are not returned within 15 days, they are called another 10 times. If provided, an additional email is sent. This procedure will be repeated if the patient has been reached yet no questionnaires are returned. If it is possible to talk to the participant at any time during the process, the most important primary outcome variables are queried. This entails that at least the most important variables can be collected, even if the participant does not return the questionnaires. As a last resort, a voluntarily provided contact of the participant is called. If none of these attempts succeed, the follow-up assessment will be terminated. Participants receive 20 CHF for each follow-up assessment.

### Procedure for substudies

#### Cortisol substudy

Inclusion and exclusion criteria for the substudy match those of the main study. The first 80 patients who are recruited in the Suedhang Hospital will be additionally asked to participate in the cortisol substudy, which involves collecting a salvia-cortisol sample before and after the first training session.

#### EEG substudy

As this involves more time-consuming measurements at T2 and T3, a two-level recruitment-process will be implemented: Inclusion and exclusion criteria for the substudy match those of the main study. However, recruitment of these participants will additionally depend on their mental capacity, age and gender balance, and their available time considering the clinic’s already demanding agenda. If eligible patients are interested in participating, additional information concerning EEG measurements will be provided, and an additional informed consent for this substudy will be signed. When 20 participants per training version are included in this substudy, the recruitment procedure will be stopped. Patients enrolled in the EEG substudy will receive a monetary compensation of 50 CHF per measurement.

### Procedure for healthy controls

Interested potential participants will receive the participant’s information and informed consent to read. If eligible for the study, a member of the study team will verify the inclusion and exclusion criteria during a screening. If a person is included in the study, written informed consent will be obtained, and the subject will undergo the same experimental tasks (IAT, GNG, SST) and a similar questionnaire battery as the patient sample at T1 (for an overview, see Additional file 5). Questionnaires and behavioral data during tasks will be collected from 30 healthy controls, the same measurement will be collected from another 20 additional healthy controls while an EEG is recorded during the experimental tasks (T2 and T3). Healthy controls without an EEG will receive no monetary compensation, whereas subjects in the EEG group will receive 30 CHF for any EEG-related inconvenience.

## Data collection and statistical analysis

### Questionnaires

Will be assessed by using paper and pencil and will then be entered into SPSS (Version 24.0, IBM SPSS Statistics for Windows, IBM Corp, Armonk, NY, USA). Behavioral data will be collected with E-Prime 2.0 (EP2Pro2.0.10.356, Psychology Software Tools, Inc., Sharpsburg, PA, USA).

### EEG data

Electrophysiological data will be recorded with BrainVision Recorder (Version 2.0, Brain Products GmbH, Gilching, Germany) using 64 active electrodes distributed across the scalp according to the extended 10/10 system. The sampling rate is 500 Hz, online filters are set to 0.016 Hz (high-pass) and 250 Hz (low pass), impedances are kept below 20 kΩ, FCz will serve as on-line reference. Each participant will first undergo a 5-min resting state EEG with alternating epochs of eyes open and eyes closed. Then, the experimental test battery of the T2 and T3 assessment, consisting of a Go-NoGo Task (GNG), an Implicit Association Test (IAT) and a Stop-Signal Task (SST), will be administered while the EEG is still recorded. For details about the tasks, please see section “Experimental tasks.”

### Cortisol data

Saliva samples for *cortisol analyses* will be stored at − 80 °C.

### Statistics

All statistical analyses will be conducted by the members of the study team. The main analyses of the training effect will be carried out after data collection is completed to maintain the study’s double-blind design. All behavioral and questionnaire variables will be tested for normal distribution (Kolmogorov-Smirnov test: *p* > 0.1, for all variables).

#### Main study

Training effects on our primary outcome measures (PDA and HDD at 3-month follow-up) will be assessed with a 2 × 3 analysis of variance (ANOVA) with the factors time point (T1, T5) and training group (Alc-IT (50/50), Alc-IT (75/25) or control). Further, a Cox regression will be computed to predict the effect of the intervention on TFD (at 3-month follow-up).

Training effects on experimental test parameters will be assessed with a 2 × 2 × 3 repeated measures ANOVA with the factors measurement point (T2, T3), time of day (morning, afternoon) and training group (Alc-IT (50/50), Alc-IT (75/25) or control). Where Mauchly’s test of sphericity indicates heterogeneity of covariance, we will verify repeated measures results with Greenhouse-Geisser corrections.

#### EEG substudy

All raw EEG data will be pre-processed with BrainVision Analyzer (Version 2.0, Brain Products GmbH, Gilching, Germany) according to current standards including ICA-based correction of eye-movement artefacts, artefact rejection and application of band-pass filtering (see e.g., [94–96]). Event-related potentials (ERPs) will be computed for each stimulus type in the three experiments (IAT, GNG, SST). Epochs from 500 ms (pre-stimulus) to 1500 ms (post-stimulus) will be averaged separately for each stimulus type and measurement point (T2 and T3). ERPs will subsequently be statistically compared for overall amplitude (i.e., global field power, GFP) and topography. For each ERP, GFP [97] will be calculated as the standard deviation across electrodes, thus measuring momentary global signal strength regardless of topographic modulations. GFP values for each point in time will be extracted and compared (T2 vs. T3) with nonparametric randomization tests, which simultaneously control for multiple comparisons. The analyses will be conducted using the Ragu software [98, 99].

To inspect for topographic differences between ERPs measured before and after the training, a topographic analysis of variance (TANOVA) [100] will be computed in Ragu. Here, dissimilarities of electric field topographies are identified with a nonparametric randomization test. A significant finding in the TANOVA indicates that activation in underlying brain structures vary in relation to the factor under investigation. Significant TANOVA results will be further explored with the standardized low-resolution electromagnetic tomography (sLORETA) source analysis method [101]) to determine which brain regions vary in activation in relation to stimulus type and/or measurement time (T2 vs. T3).

Based on prior findings, our ERP analyses will focus on the timeframe from about 150 to about 850 ms. Exact timeframes will be defined based on components as visible in GFP-curves or by microstate analyses [102, 103]. Following earlier research, care will be taken to include the N2 and P3 components in case of the GNG [25, 26] and the N2, P3, N4 and LPP components in case of the IAT (e.g., [104–106]).

#### Cortisol substudy

Salivary cortisol concentrations are determined by a commercially available chemiluminescence immunoassay (CLIA; IBL, Hamburg, Germany). Inter- and intra-assay coefficients of variation are both below 8%. For biochemical analyses of free cortisol concentration, saliva samples will be thawed and spun at 3000 rpm for 10 min to obtain 0.5–1.0 ml of clear saliva with low viscosity.

### Power calculation

The intended sample size was calculated with G*Power (Version 3.1.5, Heinrich-Heine-University Duesseldorf, Dusseldorf, Germany). In a-priori analyses (1−β = 0.8, α = 0.05), slightly reduced expected effect sizes were entered compared to earlier studies because of the additional manipulations concerning different training versions and time of training. For the 2 × 3 ANOVA to examine the training effects on the primary outcome measures PDA and HDD (with an expected effect size of f = 0.2 based on earlier studies [32]), a total sample size of *n* = 244 is needed. For the analyses of the training’s effect on TFD (the third primary outcome, estimating a small to medium effect size of w = 0.25 based on earlier studies [37], and df = 3), G*Power indicated a total sample size of *n* = 174 for the Cox regression. For the 2 × 2 × 3 repeated measures ANOVA examining training effects on experimental parameters and also taking into account the effects of time of day of the training (expecting small to medium effects of f = 0.15 and correlations of 0.4 among repeated measures based on earlier studies [107]), the necessary sample size was *n* = 180. Considering the necessary power for all analyses and similar sample sizes in all study arms (82 per intervention group), a total of 246 patients will be recruited.

## Discussion

The results of the INTRA study should provide evidence for the efficacy of an add-on treatment to specialized standard care for AUD. In contrast to previous non-clinical studies [32, 33], this double-blind RCT investigates the effect of a computerized inhibition training in inpatients with AUD. It includes important measures of alcohol consumption and follow-up periods of up to 1 year. With respect to working mechanisms, the study includes a thorough assessment of inhibitory control functions and alcohol-specific implicit associations on both a behavioral and neurophysiological level. Through this investigation, we hope to expand current knowledge about the role of inhibitory functions in AUD and extend previous findings about the effects of this training [25, 26, 32–37]. In order to describe the patients’ baseline measures, including possible inhibitory deficits, and to better interpret the observed modifications, the patients’ behavioral and neurophysiological data will be compared to healthy controls.

In additional to these proximal findings, the effect of the intervention will also be analyzed longitudinally. At 3-, 6- and 12-month follow-ups, important measures of alcohol consumption, our primary outcome, as well as psychological parameters will be collected to inform us about the time after inpatient treatment. Finally, this trial will investigate whether endogenous cortisol might increase possible effects of the inhibition training.

As one potential limitation, one could argue that patients in the control training group undergo a non-context-specific inhibition training, which might enhance their general inhibitory capacities. The choice of the control group is, therefore, very strict and could lead to an underestimation of the effect of the inhibition training. However, control conditions used in previous non-clinical studies [32, 33] consistently paired alcohol stimuli with Go responses and tended to enhance alcohol consumption in these samples. Therefore, due to ethical considerations, we opted against this control condition in a clinical sample.

Overall, the current double-blind RCT is the first study to investigate the effect of an inhibition training in an inpatient treatment setting in patients with AUD. It allows a detailed proximal and distal evaluation of behavioral, psychological and neurophysiological processes in AUD and of the efficacy of the inhibition training. We therefore hope that it might ultimately contribute to the improvement of evidenced-based AUD treatment.

## Trial status

The trial is currently in the data collection phase, which is planned to end in March 2020.

## Additional files

Additional file 1: Inclusion/exclusion criteria for all participants. (PDF 21 kb)

Additional file 2: Representative pictures of the five stimulus sets. (PDF 126 kb)

Additional file 3: Overview of Implicit Association Test (IAT) blocks for both versions. (PDF 90 kb)

Additional file 4: Standard Protocol Items: Recommendations for International Trials (SPIRIT) Checklist. (PDF 191 kb)

Additional file 5: Overview of measures for healthy controls. (PDF 18 kb)

## Data Availability

Not applicable as the current manuscript does not contain data.

## Abbreviations

AASE-G: Alcohol Abstinence Self-Efficacy Scale – German version
Alc-IT: Alcohol-inhibition training
ANOVA: Analysis of variance
ASPD: Antisocial personality disorder
ASRS-V1.1: ADHD Self Report Scale
AUD: Alcohol use disorder
AUDIT: Alcohol Use Disorders Identification Test
AUD-S: Alcohol Use Disorder Scale
BAI: Beck Anxiety Inventory
BDI-II: Beck Depression Inventory (Version 2)
BIS/BAS: Behavioral Inhibition System/Behavioral Approach System Scales
BSCL: Brief Symptom Check List
CAEQ: Comprehensive Alcohol Expectancy Scale
DIA-X: Diagnostic Expert System for Psychiatric Disorders
DSM-5: *Diagnostic and Statistical Manual of Mental Disorders* (Version 5)
DSM-IV: *Diagnostic and Statistical Manual of Mental Disorders* (Version 4)
DUDIT: Drug Use Identification Test
EEG: Electroencephalography
EOC: Errors of commission
ERP: Event-related potential
GFP: Gobal field power
GNG: Go-NoGo Task
HDD: Heavy-drinking day
HDL: Health and Daily Living Form
I-8: Scale for Impulsive Behavior
IAT: Implicit Association Test
INTRA: Inhibition training
OCDS-G: Obsessive Compulsive Drinking Scale – German version
PDA: Percentage of days abstinent
PSS: PTSD Screening Scale
SCI: Stress and Coping Inventory
SD: Standard drink
sLORETA: Standardized low-resolution electromagnetic tomography
SOCRATES: Change Readiness and Treatment Eagerness Scale
SPIRIT: Standard Protocol Items: Recommendations for International Trials
SSRT: Stop-signal reaction time
SST: Stop-Signal Task
SUD: Substance use disorder
TANOVA: Topographic analysis of variance
TFD: Time to first drink
TLFB: Timeline Follow-back
TMT: Trail Making Test
WHO: World Health Organization
WHOQOL-BREF: World Health Organization Quality of Life (brief version)
