# Supplementary material for: Learning to resist the urge: a double-blind, randomized controlled trial investigating alcohol-specific inhibition training in abstinent patients with alcohol use disorder
Source: Trials. 2019 Jul 5;20:402. doi: 10.1186/s13063-019-3505-2 (PMC6612135; doi:10.1186/s13063-019-3505-2)
Supplement: Supplementary file 1 — Inclusion/exclusion criteria for all participants. (PDF 21 kb) [file 13063_2019_3505_MOESM1_ESM.pdf]

**Additional File 1** In- and exclusion criteria for all participants

| **Inclusion criteria** | **Exclusion criteria** |
| --- | --- |
| **Patients** | |
| - Age ≥ 18 and ≤ 60 - Abstinent patients with AUD in the inpatient program of the Clinic Suedhang, the Forel Hospital, Psychiatry Hospital of Muensingen (Bern), all three are specialized centers for the treatment of addictive disorders - Abstained from alcohol for at least 4 weeks (relative to the timepoint of the beginning of the training intervention) - Informed Consent as documented by signature | - Other main psychiatric diagnosis than alcohol use disorder (comorbidity is allowed if the AUD is to be considered the main diagnosis) - Other severe substance use disorder (except nicotine; DUDIT ≥ 25 for a substance) - No known diagnosed neurocognitive problems (e.g. Korsakoff syndrome) in medical history - Current medical conditions preventing participation (e.g. acute infectious disease) - Inability to read and understand the participant’s information - Enrolment of the investigator, his/her family members, employees and other dependent persons not possible |
| **Healthy Controls** | |
| - Age ≥ 18 and ≤ 60 - Non-problematic drinking behaviour   (AUDIT < 8; AUD-S < 2)   - No psychopharmacological medication - Not currently in treatment for psychiatric diagnosis - No signs of psychopathology   (BSCL GSI_t-value_ ≤ 63)   - Informed Consent as documented by signature | - Current psychiatric diagnosis - Treatment for substance use disorders in the past - Problematic substance use (except nicotine; e.g. cannabis; DUDIT ≥ 8 for a substance) - Neurocognitive problems (e.g. Korsakoff syndrome) - Current medical conditions excluding participation (e.g. acute infectious disease) - Inability to read and understand the participant’s information |
|  | ***Only for EEG*** |
|  | - AUD in first-grade relatives (only for EEG) - Hearing impairments (only for EEG) - Current brain injury (only for EEG) |

**Legend**: AUDIT: Alcohol Use Disorders Identification Test; AUD-S: Alcohol Use Disorder Scale; BSCL: Brief Symptom Check List, EEG: Electroencephalography; GSI: Global Score Index
