# Supplementary figures and images for: Learning to resist the urge: a double-blind, randomized controlled trial investigating alcohol-specific inhibition training in abstinent patients with alcohol use disorder
Source: Trials. 2019 Jul 5;20:402. doi: 10.1186/s13063-019-3505-2 (PMC6612135; doi:10.1186/s13063-019-3505-2)

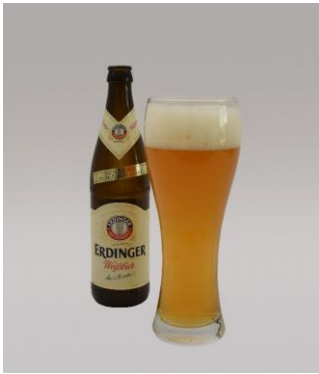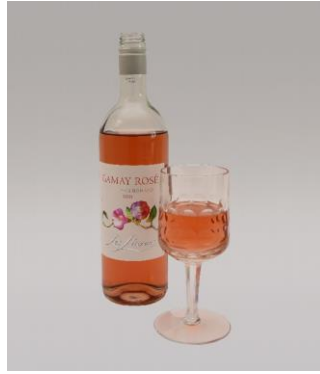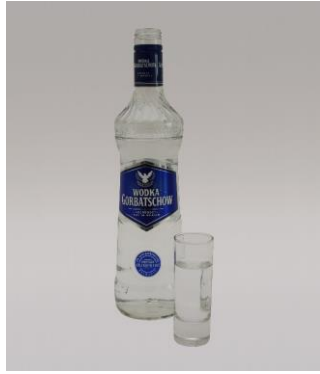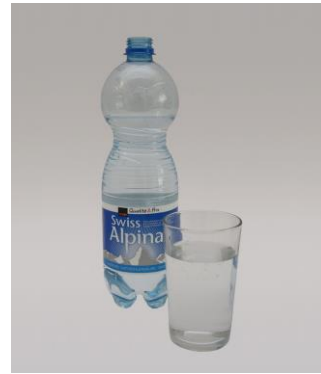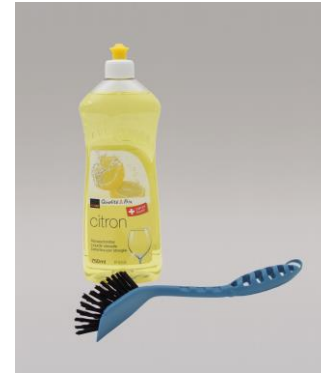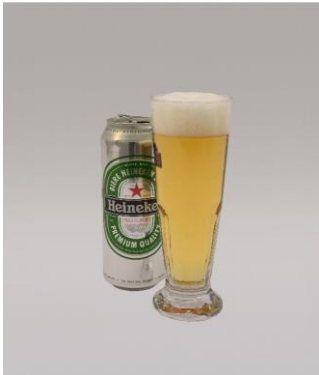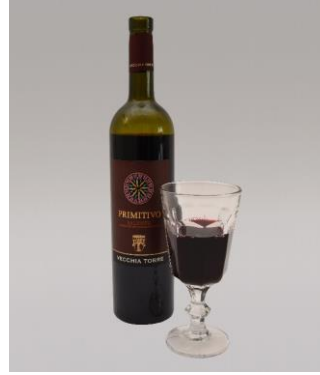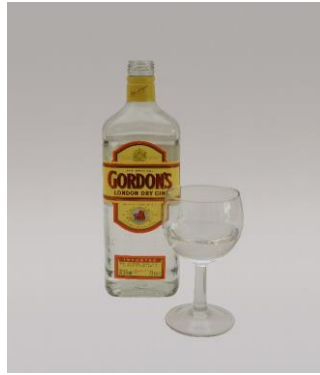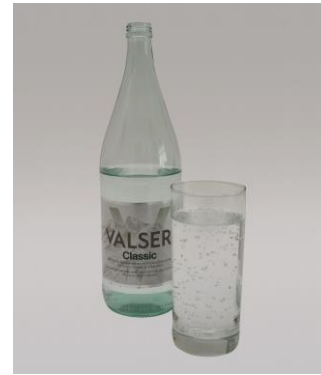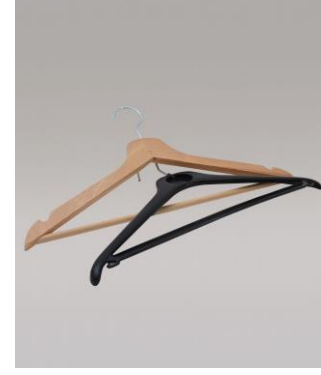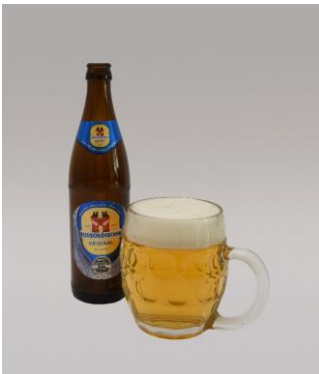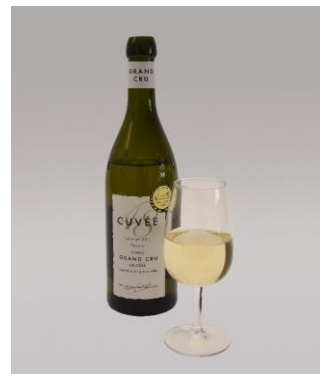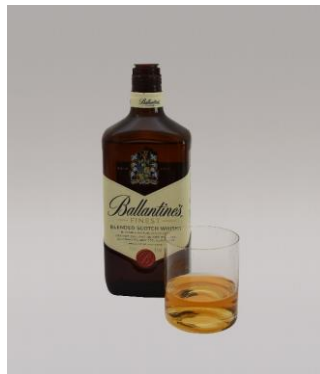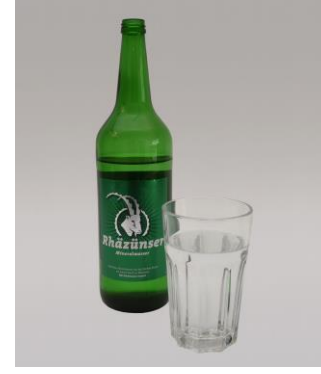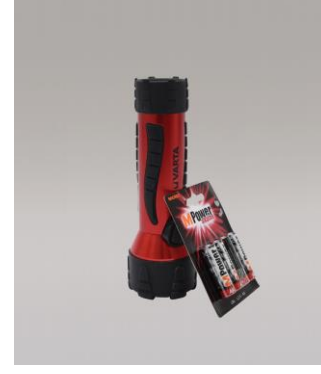

beer

wine

spirits

water

neutral

Supplement: Supplementary file 2 — Representative pictures of the five stimulus sets. (PDF 126 kb) [file 13063_2019_3505_MOESM2_ESM.pdf]
