# Supplementary material for: Learning to resist the urge: a double-blind, randomized controlled trial investigating alcohol-specific inhibition training in abstinent patients with alcohol use disorder
Source: Trials. 2019 Jul 5;20:402. doi: 10.1186/s13063-019-3505-2 (PMC6612135; doi:10.1186/s13063-019-3505-2)
Supplement: Supplementary file 3 — Overview of Implicit Association Test (IAT) blocks for both versions. (PDF 90 kb) [file 13063_2019_3505_MOESM3_ESM.pdf]

Additional File 3 Overview of IAT blocks for both versions

Block		**Version PNNP** (left label - right label)	**Version NPPN** (left label - right label)	No of Trials
B1	P(target)	alcohol - water	water - alcohol	16
B2	P(affective)	positive - negative	positive - negative	16
B3	P(combi)	alcohol/positive - water/negative	water/positive – alcohol/negative	16
B4	***Test***	*alcohol/**P**ositive - water/negative*	*water/positive – alcohol/**N**egative*	*64*
B5	P(affective)	negative - positive	negative - positive	16
B6	P(combi)	alcohol/negative - water/positive	water/negative – alcohol/positive	16
B7	***Test***	*alcohol/**N**egative - water/positive*	*water/negative – alcohol/**P**ositive*	*64*
B8	P(target)	water - alcohol	alcohol - water	16
B9	P(affective)	positive - negative	positive - negative	16
B10	P(combi)	water/positive – alcohol/negative	alcohol/positive-water/negative	16
B11	***Test***	*water/positive – alcohol/**N**egative*	*alcohol/**P**ositive – water/negative*	*64*
B12	P(affective)	negative - positive	negative - positive	16
B13	P(combi)	water/negative – alcohol/positive	alcohol/negative - water/positive	16
B14	***Test***	*water/negative – alcohol/**P**ositive*	*alcohol/**N**egative - water/positive*	*64*

Legend: P: practice; B: block
