# Supplementary material for: Learning to resist the urge: a double-blind, randomized controlled trial investigating alcohol-specific inhibition training in abstinent patients with alcohol use disorder
Source: Trials. 2019 Jul 5;20:402. doi: 10.1186/s13063-019-3505-2 (PMC6612135; doi:10.1186/s13063-019-3505-2)
Supplement: Supplementary file 4 — Standard Protocol Items: Recommendations for International Trials (SPIRIT) Checklist. (PDF 191 kb) [file 13063_2019_3505_MOESM4_ESM.pdf]

SPIRIT

STANDARD PROTOCOL ITEMS: RECOMMENDATIONS FOR INTERVENTIONAL TRIALS

SPIRIT 2013 Checklist: Recommended items to address in a clinical trial protocol and related documents*

Section/item	Item No	Description	Addressed on page number
**Administrative information**			m = manuscript<br>s = SPIRIT
Title	1	Descriptive title identifying the study design, population, interventions, and, if applicable, trial acronym	1 (m)
Trial registration	2a	Trial identifier and registry name. If not yet registered, name of intended registry	2 (m)
	2b	All items from the World Health Organization Trial Registration Data Set	7, 23, 24 (m)
Protocol version	3	Date and version identifier	6 (s)
Funding	4	Sources and types of financial, material, and other support	24 (m)
Roles and responsibilities	5a	Names, affiliations, and roles of protocol contributors	1 (m), 7(s)
	5b	Name and contact information for the trial sponsor	1 (m)
	5c	Role of study sponsor and funders, if any, in study design; collection, management, analysis, and interpretation of data; writing of the report; and the decision to submit the report for publication, including whether they will have ultimate authority over any of these activities	24f (m)
	5d	Composition, roles, and responsibilities of the coordinating centre, steering committee, endpoint adjudication committee, data management team, and other individuals or groups overseeing the trial, if applicable (see Item 21a for data monitoring committee)	25 (m)

**Introduction**			
Background and rationale	6a	Description of research question and justification for undertaking the trial, including summary of relevant studies (published and unpublished) examining benefits and harms for each intervention	3ff (m)
	6b	Explanation for choice of comparators	10f (m)
Objectives	7	Specific objectives or hypotheses	7f (m)
Trial design	8	Description of trial design including type of trial (eg, parallel group, crossover, factorial, single group), allocation ratio, and framework (eg, superiority, equivalence, noninferiority, exploratory)	7 (m)
**Methods: Participants, interventions, and outcomes**			
Study setting	9	Description of study settings (eg, community clinic, academic hospital) and list of countries where data will be collected. Reference to where list of study sites can be obtained	7 (m)
Eligibility criteria	10	Inclusion and exclusion criteria for participants. If applicable, eligibility criteria for study centres and individuals who will perform the interventions (eg, surgeons, psychotherapists)	8f (m)
Interventions	11a	Interventions for each group with sufficient detail to allow replication, including how and when they will be administered	10f (m)
	11b	Criteria for discontinuing or modifying allocated interventions for a given trial participant (eg, drug dose change in response to harms, participant request, or improving/worsening disease)	24 (m)
	11c	Strategies to improve adherence to intervention protocols, and any procedures for monitoring adherence (eg, drug tablet return, laboratory tests)	18 (m)
	11d	Relevant concomitant care and interventions that are permitted or prohibited during the trial	not applicable
Outcomes	12	Primary, secondary, and other outcomes, including the specific measurement variable (eg, systolic blood pressure), analysis metric (eg, change from baseline, final value, time to event), method of aggregation (eg, median, proportion), and time point for each outcome. Explanation of the clinical relevance of chosen efficacy and harm outcomes is strongly recommended	12ff (m)
Participant timeline	13	Time schedule of enrolment, interventions (including any run-ins and washouts), assessments, and visits for participants. A schematic diagram is highly recommended (see Figure)	17ff (m)

Sample size	14	Estimated number of participants needed to achieve study objectives and how it was determined, including clinical and statistical assumptions supporting any sample size calculations	22 (m)
Recruitment	15	Strategies for achieving adequate participant enrolment to reach target sample size	7f (m)

Methods: Assignment of interventions (for controlled trials)

Allocation:			
Sequence generation	16a	Method of generating the allocation sequence (eg, computer-generated random numbers), and list of any factors for stratification. To reduce predictability of a random sequence, details of any planned restriction (eg, blocking) should be provided in a separate document that is unavailable to those who enrol participants or assign interventions	18 (m)
Allocation concealment mechanism	16b	Mechanism of implementing the allocation sequence (eg, central telephone; sequentially numbered, opaque, sealed envelopes), describing any steps to conceal the sequence until interventions are assigned	18 (m)
Implementation	16c	Who will generate the allocation sequence, who will enrol participants, and who will assign participants to interventions	18 (m)
Blinding (masking)	17a	Who will be blinded after assignment to interventions (eg, trial participants, care providers, outcome assessors, data analysts), and how	18 (m)
	17b	If blinded, circumstances under which unblinding is permissible, and procedure for revealing a participant's allocated intervention during the trial	18 (m)

Methods: Data collection, management, and analysis

Data collection methods	18a	Plans for assessment and collection of outcome, baseline, and other trial data, including any related processes to promote data quality (eg, duplicate measurements, training of assessors) and a description of study instruments (eg, questionnaires, laboratory tests) along with their reliability and validity, if known. Reference to where data collection forms can be found, if not in the protocol	11ff (m)
	18b	Plans to promote participant retention and complete follow-up, including list of any outcome data to be collected for participants who discontinue or deviate from intervention protocols	19 (m)

Data management	19	Plans for data entry, coding, security, and storage, including any related processes to promote data quality (eg, double data entry; range checks for data values). Reference to where details of data management procedures can be found, if not in the protocol	19 (m)
Statistical methods	20a	Statistical methods for analysing primary and secondary outcomes. Reference to where other details of the statistical analysis plan can be found, if not in the protocol	20f (m)
	20b	Methods for any additional analyses (eg, subgroup and adjusted analyses)	21f (m)
	20c	Definition of analysis population relating to protocol non-adherence (eg, as randomised analysis), and any statistical methods to handle missing data (eg, multiple imputation)	14 (s)
**Methods: Monitoring**			
Data monitoring	21a	Composition of data monitoring committee (DMC); summary of its role and reporting structure; statement of whether it is independent from the sponsor and competing interests; and reference to where further details about its charter can be found, if not in the protocol. Alternatively, an explanation of why a DMC is not needed	16 (s)
	21b	Description of any interim analyses and stopping guidelines, including who will have access to these interim results and make the final decision to terminate the trial	16 (s)
Harms	22	Plans for collecting, assessing, reporting, and managing solicited and spontaneously reported adverse events and other unintended effects of trial interventions or trial conduct	18f (m)
Auditing	23	Frequency and procedures for auditing trial conduct, if any, and whether the process will be independent from investigators and the sponsor	16 (s)
**Ethics and dissemination**			
Research ethics approval	24	Plans for seeking research ethics committee/institutional review board (REC/IRB) approval	24 (m)
Protocol amendments	25	Plans for communicating important protocol modifications (eg, changes to eligibility criteria, outcomes, analyses) to relevant parties (eg, investigators, REC/IRBs, trial participants, trial registries, journals, regulators)	24 (m)

Consent or assent	26a	Who will obtain informed consent or assent from potential trial participants or authorised surrogates, and how (see Item 32)	17ff (m)
	26b	Additional consent provisions for collection and use of participant data and biological specimens in ancillary studies, if applicable	19f (m)
Confidentiality	27	How personal information about potential and enrolled participants will be collected, shared, and maintained in order to protect confidentiality before, during, and after the trial	17 (s)
Declaration of interests	28	Financial and other competing interests for principal investigators for the overall trial and each study site	25 (m)
Access to data	29	Statement of who will have access to the final trial dataset, and disclosure of contractual agreements that limit such access for investigators	17 (s)
Ancillary and post-trial care	30	Provisions, if any, for ancillary and post-trial care, and for compensation to those who suffer harm from trial participation	17 (s)
Dissemination policy	31a	Plans for investigators and sponsor to communicate trial results to participants, healthcare professionals, the public, and other relevant groups (eg, via publication, reporting in results databases, or other data sharing arrangements), including any publication restrictions	17 (s)
	31b	Authorship eligibility guidelines and any intended use of professional writers	17 (s)
	31c	Plans, if any, for granting public access to the full protocol, participant-level dataset, and statistical code	17 (s)
**Appendices**			
Informed consent materials	32	Model consent form and other related documentation given to participants and authorised surrogates	18 (s)
Biological specimens	33	Plans for collection, laboratory evaluation, and storage of biological specimens for genetic or molecular analysis in the current trial and for future use in ancillary studies, if applicable	22 (m)

*It is strongly recommended that this checklist be read in conjunction with the SPIRIT 2013 Explanation & Elaboration for important clarification on the items. Amendments to the protocol should be tracked and dated. The SPIRIT checklist is copyrighted by the SPIRIT Group under the Creative Commons "Attribution-NonCommercial-NoDerivs 3.0 Unported" license.

SPIRIT 2013 Checklist: Recommended items to address in a clinical trial protocol and related documents*

Section/item	Item No	Description
**Administrative information**		
Title	1	Learning to resist the urge: Inhibition training in abstinent patients with alcohol use disorder. A double-blind randomized controlled trial (INTRA).
Trial registration	2a	Primary registration: ClinicalTrials.gov NCT02968537<br>Secondary identifying number: Swiss National Clinical Trails Portal SNCTP000002043 (see page 2 in manuscript).
	2b	Date of registration: November 10, 2016<br>Date of first enrolment: December 2016<br>Allocation: Randomized<br>Intervention Model: Parallel Assignment<br>Masking: Double-blind<br>Primary Purpose: Treatment<br>Recruitment status: Recruiting main study
Protocol version	3	Patients: Version 3.2 of January 24, 2018<br>Healthy controls: Version 3.4_localA of May 25, 2018<br>*Revision chronology*<br>Version 1: Original<br>Version 2 (2016-Jul-8): Specification recruitment sub-study EEG<br>Version 2.1 (2016-Nov-29): Addition of questionnaires (traits)<br>Version 3.0 (2017-Jun-6): Addition healthy control (behavioral)<br>Version 3.2 (2018-Jan-24): Addition study-side Muensingen<br>Version 3.4 (2018-Jun-5): Addition healthy control (EEG)
Funding	4	Grant from the Swiss National Foundation (SNF; No: 105319_159286) (see page 24 in manuscript).
	5a	Maria Stein[1,3]; Principal Investigator and Sponsor Investigator<br>Leila M. Soravia[1,2]; (Local) Principal Investigator<br>Franz Moggi[1]; Principal Investigator<br>Hallie M. Batschelet[1]; Investigator, PhD student<br>Raphaela M. Tschuemperlin[1,2]; Investigator, PhD student<br><br>[1] University Hospital of Psychiatry and Psychotherapy Bern, Translational Research Center, Bolligenstrasse 111, CH-3000 Bern 60, Switzerland.

[2] Clinic Suedhang, Center for the treatment of addictive disorders, Südhang 1, CH-3038 Kirchlindach, Switzerland.
[3] University of Bern, Institute for Psychology, Fabrikstrasse 8, CH-3012 Bern, Switzerland (see page 1 in manuscript).

5b University Hospital of Psychiatry and Psychotherapy
Translational Research Center
Bolligenstrasse 111
CH-3000 Bern 60. Switzerland
Project leader: Dr. phil. Maria Stein
Phone: +41 (0)31 930 87 16
Fax : +41 (0)31 930 99 61
Email : maria.stein@upd.unibe.ch (see page 1 in manuscript).

5c The funding sources had no role in the design of this study and will not have any role in its recruitment, analyses, interpretation or the decision to submit results (see page 24f in manuscript).

5d **Principal investigator(s)**

- Design development and funding of the trial
- Agreeing with the blinder on the developed procedure
- Supervision and reviewing of the study process
- Budget administration
- Supervision of data management
- Data analyses and supervision of study members conducting data analyses

Local principal investigators

- Supporting implementation (e.g. informing employees about cooperation, establishes contact between PhD students and co-workers)
- Supporting recruitment at study sites

PhD students

- Implementation of the design
- Recruitment and data collection at the three study sites
- Data monitoring (supervision of data entry, CRF)
- Data analyses

Principal investigators and PhD students

- Preparation of the ethic approval (including IC, CRF) and the study protocol
- Publication of data

Independent researcher

- Randomization

(see page 25 in manuscript)

Introduction

Background and rationale	6a	Therapeutic interventions to treat alcohol use disorder (AUD) still need to be improved. Most current conceptualizations of AUD postulate enhanced automatic reaction to alcoholic cues and impaired inhibitory control as relevant processes in the development, maintenance, and treatment of AUD. Complementing traditional cognitive behavioral relapse prevention strategies, novel computerized training interventions aim to directly interfere with implicit processes involved in addictive behaviors. For three reasons, a novel alcohol-specific inhibition-training seems to be a promising new intervention: First, this training intervention effectively reduced drinking behavior and implicit attitudes towards alcohol in a heavy drinking student population [1, 2]. Second, recent neurophysiological findings suggest that alcohol-specific inhibition (tested with Go-NoGo tasks in an alcohol-related context) demand additional neuronal resources in AUD patients. Such a training thus recruits a network which is probably impaired in AUD patients [3, 4]. According to these findings, an alcohol-specific inhibition-training targets crucial neuronal processes, whose improvement may lead to modification of highly relevant clinical variables. Third, this short, computerized training is very easy to implement in inpatient and outpatient settings as neither expensive equipment nor specially trained staff is needed. It might thus be an adequate and cost-effective add-on to traditional treatment and may enhance clinical outcome of AUD. The proposed study now tests this training for the first time in a patient population. In this study, recently abstinent patients with AUD attending an inpatient treatment program will be randomly assigned to one of two alcohol-specific inhibition-training groups (varying in Go/NoGo-ratio) or to a control condition. Besides the effects on drinking behavior, inhibition and implicit attitudes, we expect the training to influence neurophysiological reactivity to alcohol related stimuli. A subgroup of patients will therefore additionally undergo EEG recording so that the neurophysiological effects of the training can be assessed and related to clinical outcome. To compare patients' data, 40 healthy controls will be measured behaviourally at one point in time and 20 healthy controls will be measured with EEG at one point of time. Since training effects rely on learning processes, the influence of endogenous cortisol level (a consolidation–enhancer which peaks in the morning) on training outcome will be examined by the variation of daytime of the training. All patients' inhibitory control and implicit associations towards alcohol will be measured before and after training. The training effects will be examined on proximal outcome variables (e.g. implicit associations, inhibitory control, abstinence related self-efficacy, craving) and on distal outcome variables at 3-, 6- and 12-months follow-up (e.g. percent abstinent days (primary outcome)). For

		the first time the planned project will investigate the therapeutic potential of an alcohol-specific inhibition-training as an add-on in a patient population. In doing so, it will additionally investigate the impact of variables such as daytime and Go/NoGo-ratio on training efficacy and elucidate the underlying neurophysiological mechanisms of the training. The project thus aims to contribute to the improvement of evidence-based treatment of AUD (see page 3ff in manuscript).
	6b	**Control-training:** This control-training is thus of equal lengths as the other two groups and has the same number of alcohol-related pictures in it. But all pictures will now be combined in equal probabilities with a Go-response and a NoGo-response. The control condition thus consists of an unspecific inhibition training. We are aware that this is a strong control condition, but for ethical reasons we opted against the use of Alc-Goes in an AUD population. As in the other two groups, patients will be given Feedback about their performance (number of errors and mean reaction time) after each training session (see page 10f in manuscript).
Objectives	7	The primary goal of this study is to examine the effects of an alcohol-specific inhibition-training (Alc-IT) on drinking behavior (PDA, HDD, TFD, see research questions in manuscript) in a patient population: Does the training lead to an improvement of the standard-treatment in abstinent patients with AUD?<br>Secondary objectives concern the effects of the Alc-IT on psychological and experimental parameters (see research questions in main manuscript) and will help to elucidate working mechanisms of Alc-IT (see page 7f in manuscript).
Trial design	8	Double-blind, randomized, controlled trial (see page 7 in manuscript).

Methods: Participants, interventions, and outcomes

Study setting	9	Data will be collected in three specialized addiction treatment centres in Switzerland: The Suedhang Hospital (Bern), the Forel Hospital (Zuerich), and the Psychiatric Hospital of Muensingen (Bern). All eligible patients of a specialized inpatient treatment program will be asked for participation. These inpatient programs vary in duration and interventions, but all focus on the treatment of AUD and are lasting several weeks (see page 7 in manuscript).
Eligibility criteria	10	***Patients (main study and sub-studies cortisol and EEG;*** see page 8f in manuscript)<br>Inclusion criteria:<br>• Age ≥ 18 and ≤ 60,<br>• Abstinent patients with an alcohol use disorder in the inpatient program of the Suedhang Hospital, the Forel Hospital, Psychiatry Hospital of Muensingen, all three are specialized centers for the treatment of addictive disorders,

- Abstained from alcohol for at least 4 weeks prior to the training sessions,
- Informed Consent as documented by signature.

Exclusion criteria:

- Other main psychiatric diagnosis than alcohol use disorder (comorbidity is allowed if the AUD is to be considered the main diagnosis),
- Other severe substance use disorder (except nicotine; DUDIT ≥ 25 for a substance),
- No known neurocognitive problems (e.g. Korsakoff syndrome)
- Current medical conditions preventing participation (e.g. acute infectious disease),
- Inability to read and understand the participant's information
- Enrolment of the investigator, his/her family members, employees and other dependent persons.

Healthy Controls (see page 9 in manuscript)
Inclusion criteria:

- Age ≥ 18 and ≤ 60,
- Non-problematic drinking behavior (AUDIT < 8; AUD-S < 2),
- No psychopharmacological medication,
- Not currently in treatment for psychiatric diagnosis,
- No signs of psychopathology (BSCL: $GSI_{t\text{-value}} \leq 63$),
- Informed Consent as documented by signature.

Exclusion criteria:

- Current psychiatric diagnosis,
- Treatment for substance use disorders in the past,
- Problematic substance use (except nicotine; e.g. cannabis; DUDIT ≥ 8 for a substance),
- Neurocognitive problems (e.g. Korsakoff syndrome),
- Current medical conditions excluding participation (e.g. acute infectious disease),
- Inability to read and understand the participant's information.

Only for EEG

- AUD in first-grade relatives (only for EEG),
- Hearing impairments (only for EEG),
- Current brain injury (only for EEG).

Interventions | 11a | During all training conditions, pictures of alcoholic beverages, non-alcoholic beverages or neutral control pictures will be presented on a computer screen. Two letters displayed randomly in one of the four corners of the picture will serve as Go- respectively NoGo-cue. Assignment of instruction (Go, respectively NoGo) to letters ("p" and "f") will be counterbalanced. During each trial, pictures and cues will

be displayed together on screen for 1500ms, followed for 500ms by a feedback consisting of either a green circle for correct (non)responses or a red cross for incorrect (non)responses. All three training versions will take about 13 minutes to complete. During all three versions 320 pictures (80 of alcoholic beverages, 80 of non-alcoholic beverages and 160 neutral objects) will be shown. The versions will only differ in the probability in which these pictures are combined with the Go or NoGo-cues.

During both versions of the Alc-inhibition-training, pictures of alcoholic beverages will be consistently paired with a NoGo-cue, while Go-cues will be distributed among the other two picture types.

- Alc-inhibition-training (50/50): The first training group will operate with a Go/NoGo ratio of 50/50 as in previous studies [1, 2]: This original version of the Alc-inhibition-training will include 80 alcoholic NoGo trials as well as 80 non-alcoholic Go trials. To arrive at the same training length while keeping the number of Alc-NoGo-pairings constant and the Go/NoGo-ratio at 50/50, it will additionally include 80 neutral Go-trials as well as 80 neutral NoGo-trials.

- Alc-inhibition-training (75/25): The second version of the Alc-inhibition-training will operate with a Go/NoGo-ratio of 75/25. It will equally include 80 Alcoholic NoGo-trials and 80 non-alcoholic Go-trials, but now 160 neutral Go-trials will complete the set.

- Control training: The third group will receive a control-training that consists of 80 non-alcoholic trials, 80 alcoholic trials as well as 80 neutral Go-trials and 80 neutral NoGo-trials. This control-training is thus of equal lengths as the other two groups and has the same number of alcohol-related pictures in it. But all pictures will now be combined in equal probabilities with a Go-response and a NoGo-response.

For an overview of the interventions see page 10f in manuscript.

11b If the intervention leads to burden, all participants can cancel at any point of time during each training session. They are free to continue on another day or to terminate their study participation (see page 24 in manuscript).

11c Adherence to the training protocol will be monitored by the study member present during training sessions. Furthermore, to enhance participants motivation to perform at their best possible level, patients will be given feedback about their performance (number of errors and mean reaction time) after each training session. Both performance indicators will be entered in a feedback form to provide an overview of

		the progress during the six sessions (see page 18 in manuscript).
	11d	Not applicable as all patients will receive the same specialized inpatient treatment in one of the three study sites.
Outcomes	12	**Primary outcomes (research question 1)** are percent days abstinent (PDA), heavy drinking days (HDD) and/or extend the time to first drink (TFD) in 3-months follow-up. This will be measured with the Timeline Followback (TLFB), which is a validated procedure to aid recall of past drinking and evaluates information about drinking behavior (see page 12 in manuscript).<br><br>**Secondary outcomes (research questions 2-5):**<br>2) Training effects on experimental parameters will also be investigated with validated tasks that have been used before in similar research: Inhibition will be assessed with the Stop-Signal-Task (SST) and the Go-NoGo-task (GNG), implicit associations towards alcohol are assessed with the Implicit Association Task (IAT; see page 14ff in manuscript).<br><br>3) The proposed study also aims to capture training-induced changes in neurophysiological correlates of inhibitory functions and implicit associations towards alcohol, which will be assessed with event related potentials (ERPs; see page 21 in manuscript).<br><br>4) As training initiates a learning process, we want to investigate whether the consolidation enhancing effects of endogenous cortisol may be used to increase training effects. Making use of the naturally occurring cortisol secretion pattern (with a cortisol peak in the morning), daytime of training will also be varied in order to test whether high cortisol levels during training increase outcome.<br><br>5) Psychological parameters: Training effects on abstinence related self-efficacy, craving and motivation will be assessed. Both outcomes are assessed with validated questionnaires: Self-efficacy will be assessed with the Alcohol Abstinence Self-Efficacy scale (AASE-G), craving with the Obsessive Compulsive Drinking Scale (OCDS-G). Motivation will be assessed with the Stages of Change Readiness and Treatment Eagerness Scale (SOCRATES; see page 12f in manuscript).
Participant timeline	13	**T1 Baseline**: Psychological parameters (questionnaires & scales), diagnostic interviews (DIA-X, TLFB)<br><br>**T2 Pre-training**: Questionnaires and three experimental tests (IAT, GNG, SST)<br><br>Sub-study EEG: 60 patients (20 in each of the three training versions) will additionally be assessed with multichannel EEG while participating the experimental tasks during T2 and T3 assessment.<br><br>**Inhibition training (*intervention*)**: 6 training sessions (Alc-IT 50/50 /

		Alc-IT 75/25 / control training) distributed over two weeks either in the morning or afternoon<br><br>Sub-study Cortisol: For the first 80 patients of the Clinic Südhang. analyses of saliva cortisol before and after the first training will be performed (to monitor differences in endogenous cortisol level and reactivity).<br><br>**T3 Post-training**: Questionnaires and three experimental tasks (IAT, GNG, SST) as in T2.<br><br>Sub-study EEG: As for T2, the same 60 patients (20 in each of the three training versions) will additionally be assessed with multichannel EEG while participating the experimental tasks during T3 assessment<br><br>**T4 Discharge**: Psychological parameters (questionnaires & scales).<br><br>**T5 3-months follow-up**: Psychological parameters (questionnaires & scales) send to private address after phone call, telephone interview.<br><br>**T6 / T7 6- and 12 months follow-up**: Psychological parameters (questionnaires & scales) send to private address after phone call.<br><br>For a schematic overview of the time schedule see Figure 1, for a summary of the measurements see Figure 2 (SPIRIT Figure) in manuscript. For a detailed description of the procedure see page 17ff in manuscript.<br><br>Healthy controls<br>Healthy controls will be screened for inclusion and then measured once after that with questionnaires and experimental tasks.<br>At screening, they will receive the following questionnaires: AUD-S, AUDIT, BSCL, some questions regarding possible exclusion criteria (e.g. current psychiatric treatment, brain injuries).<br><br>After inclusion, they will receive questionnaires (see Additional File 5) and perform experimental tasks: IAT, GNG, SST.<br>During experimental task, behavioural data will be collected from 40 healthy controls. 20 additional healthy controls will also undergo EEG-recording during the experimental tasks. EEG recordings will be performed in the EEG-lab in the Suedhang Hospital or at the University of Berne. For a description of the procedure see page 20 in manuscript.
Sample size	14	246 participants will be enrolled (136 at Suedhang Hospital, 75 at Forel Hospital, 35 at Psychiatry Hospital of Muensingen) and randomly assigned to one of the three study arms (82 per study arm). This sample size is justified by a power analysis (see page 22 in manuscript).
Recruitment	15	The recruitment process is embedded in the hospital surroundings of each study site. All eligible patients of the specialized long-term treatment program will receive the study information and will be invited

		for an individual information meeting, where the main-study (or the sub-study cortisol for the first 80 patients of the Suedhang Hospital) will be explained in detail.<br>To recruit the 60 patients for the sub-study EEG, all eligible patients will be asked upon inclusion in the main study whether they are interested to participate in additional EEG-measurements (see page 7f in manuscript).

Methods: Assignment of interventions (for controlled trials)

Allocation:

Sequence generation	16a	The randomization, stratified according to age and gender, will be done by an independent person, who will be the only one with access to the computer-generated stratified randomization list (see page 18 in manuscript).
Allocation concealment mechanism	16b	After a new participant will be recruited, a study member will write an email to the blinder, containing information about gender and age. The blinder will then allocate the new participant to a study arm Alc-IT 50/50, Alc-IT 75/25, control group) and to daytime of training (morning, afternoon) by following the stratified randomization list and assigning a number to the participant (see page 18 in manuscript)..
Implementation	16c	The enrolment of participants will be implemented by a member of the study team. At the study sites Forel Hospital and Psychiatry Hospital of Muensingen an investigator will organize the procedure, for the Clinic Südhang, an investigator is responsible. The assignment of participants to the intervention group will be done according to numbers, randomly allocated over the conditions (group, daytime) and allocated by the blinder, who will be the only one with access to the randomization list (see page 18 in manuscript).
Blinding (masking)	17a	During the study, participants will start each of their training sessions independently at a computer, where entering their study number will automatically lead to the presentation of the training version identified in the randomization list. Thus, care providers, data collectors, data analysts and investigators as well as the trial participants will be blind to the allocation schedule (see page 18 in manuscript).
	17b	The independent researcher is the only person having access to the randomization list. Unblinding is permissible in case of suspension, premature study termination, exclusion from the study, or study drop out. If unblinding has to be done, the independent researcher will communicate the participants allocated intervention to the investigator or members of the study team (see page 18 in manuscript).

Methods: Data collection, management, and analysis

Data collection methods	18a	Validated questionnaires, interviews and experimental tasks will be used. They will be implemented by trained study members and will be systematically checked after completion. For a description of the measurements see section *Interviews and Questionnaires* (page 11ff) in manuscript.
	18b	To encourage high response-rates at 3-, 6- and 12-months follow-up, a tight contact-procedure is planned. A member of the study team will start with telephone calls 10 days in advance of the planned date. At the first contact, the most essential information will be obtained in a short telephone-interview. In case of missing questionnaire-batteries, three sessions of telephone-contact, during which participants are called 10 times, are planned. For each returned questionnaire-battery, 20 CHF will be paid (see page 19 in manuscript).<br>In case of discontinuation, no additional data will be collected.
Data management	19	We will use a paper-based system for data management except for EEG, experimental data and clinic-intern electronically recorded questionnaires. The data will be entered into SPSS by a member of the study team according to a carefully prepared data-handling protocol. The data entry will be monitored by HB and RT (see page 20 in manuscript).
Statistical methods	20a	**Main study.** Training effects on our primary outcome measures (PDA and HDD at 3-months follow-up) will be assessed with a 2 x 3 ANOVA with the factors timepoint (T1, T5) and training group (Alc-IT (50/50), Alc-IT (75/25), control). Further, a Cox regression will be performed to predict the effect of the intervention to TFD (at 3-months follow-up).<br>Training effects on experimental test parameters will be assessed with 2 x 2 x 3 repeated measures ANOVA with the factors measurement point (T2, T3), daytime (morning, afternoon) and training group (Alc-IT (50/50), Alc-IT (75/25), control). Where the Mauchly's test of sphericity indicated heterogeneity of covariance, we will verify repeated measures results with Greenhouse-Geisser corrections (see page 20f in manuscript).
	20b	**Sub-study EEG:** All EEG raw-data will be pre-processed according to up-to-date standards (see manuscript). Then, event-related potentials (ERPs) will be computed for each stimulus type and time-point of the two three experiments (IAT, GNG, SST). Then, ERPs will be statistically compared regarding overall amplitude (i.e. Global field power, GFP) and topography (see page 21 in manuscript).<br><br>**Sub-study Cortisol:** Salivary cortisol concentrations are determined by a commercially available chemiluminescence immunoassay (CLIA; IBL, Hamburg, Germany). Inter- and intra-assay coefficients of variation are both below 8% (see page 22 in manuscript).

	20c	As strategies are implemented to prevent missing data, only few missing data are expected. For missings, including subjects who dropped out during the trial, an intention-to-treat analysis as well as multiple imputations will be performed.
**Methods: Monitoring**		
Data monitoring	21a	The trial will be monitored internally on a regular basis (meetings at least once a month to evaluate the progress of the study, and to ensure protocol requirements, regulations and obligations are being fulfilled correctly). Moreover, data will be accessible to monitors of the local authorities and questions will be answered during such a monitoring.
	21b	No interim analyses will be performed, as in this minimal-risk trial no serious events are expected.
Harms	22	There is no known risk for the measures and procedures included in this study (EEG-recording, saliva cortisol, questionnaires). The alcohol-pictures presented during the experimental tasks and the inhibition training could lead to some strain and restlessness. Therefore, we will measure stress and craving with a 11-point Likert scale before and after each training session / at pre-(T2) and post-training (T3). Moreover, all participants are in an inpatient program, with care professionals available all day. In case of acute stress and raised craving after a training session / T2 / T3, we will contact the participant and a psychiatrist on duty will be available if further help is needed (see page 18f in manuscript).
Auditing	23	No external audits are planned. However, a member of the study team will oversee the ongoing process during inpatient treatment (T1-T4) any time. Additionally, regular meetings of the study team (at least once a month) guarantee the integrity and adherence of the trial.
**Ethics and dissemination**		
Research ethics approval	24	Ethic approval of the trial was given on the 3th of October 2016 by the local Ethics Committees (EC) of Bern and Zürich. Annually progress reports are required and will be done by the PI. They will be reviewed and approved by the EC bodies (see page 24 in manuscript).
Protocol amendments	25	In case of significant changes in the protocol, formal amendments will be performed. Administrative and minor changes of the protocol will be communicated in the progress report (see page 24 in manuscript).
Consent or assent	26a	Few hours after treatment entry, information sheets will be handed over to patients. At least one day after, an information meeting will be organized. Trained study members will introduce the study to patients in a face-to-face setting, where the trial got explained and questions answered. After the informed discussion written informed consent will be obtained (see page 17 in manuscript).

		For the sub-study healthy controls possible participants will be informed face-to-face, receive the study information. In a first meeting they give informed consent and fill out the screening questionnaires. All participants receive a copy of their informed consent shortly before the first measurement starts (see page 20 in manuscript).
	26b	The first 80 patients who are recruited in the Suedhang Hospital will be asked for two salvia samples (sub-study Cortisol). They receive a separate study information and informed consent including details of the biological samples (see page 19 in manuscript). For the sub-study *EEG*, the two-step-recruitment follows after inclusion in the main study. After the first measurement, they obtain second information sheets and informed consents with marked additional details. Therefore, a second written informed consent is needed (see page 19f in manuscript).
Confidentiality	27	All study-relevant information will be stored coded at each study site. During data acquisition, the code list will be administrated electronically and will be saved on a secured electronic platform provided by each study site. These lists will be password-protected and will therefore only be accessible for the study team. After data acquisition, the code list will be kept under lock and key and will be stored in a single file in a fire-proof safe at the Translational Research Center, University Hospital of Psychiatry, Bolligenstrasse 111, CH-3000 Bern 60. Only members of the study-team in need of this information will have access to it.
Declaration of interests	28	No declaration of interest must be declared (see page 25 in manuscript).
Access to data	29	All Principal Investigators and Investigators will be given access to cleared data sets of all three study sites.
Ancillary and post-trial care	30	This is a category A study (Clinical trials with interventions that are neither a therapeutic product nor a transplant product, nor a transplant) according to the Swiss law on human research. Therefore, no specific study insurance is needed.
Dissemination policy	31a	Study results will be published in scientific journals and will be assimilated at scientific conferences. If participants sign a form, indicating interest in results, they will get a summary of the most important findings.
	31b	Principal Investigators and Investigators will write scientific articles. No professional writers are involved.
	31c	Requests about public access to the full protocol or statistical codes will be evaluated by the Principal Investigators.

Appendices

Informed consent materials	32	In the appendix, the latest versions of informed consent material are provided:<br>• Patient Informed Consent main study<br>• Patient Informed Consent sub-study cortisol<br>• Patient Informed Consent sub-study EEG<br>• Informed Consent healthy control<br>• Informed Consent healthy control EEG
Biological specimens	33	Salvia samples will be stored until the end of the data collection in a monitored freezer. Afterwards, they will be analysed at University of Dresden. Results of the analysis of the cortisol samples will be send from the University of Dresden to the responsible investigator LS with numeric codes (see page 22 in manuscript).

*It is strongly recommended that this checklist be read in conjunction with the SPIRIT 2013 Explanation & Elaboration for important clarification on the items. Amendments to the protocol should be tracked and dated. The SPIRIT checklist is copyrighted by the SPIRIT Group under the Creative Commons "Attribution-NonCommercial-NoDerivs 3.0 Unported" license.

1. Houben K, Havermans RC, Nederkoorn C, Jansen A. Beer a no-go: learning to stop responding to alcohol cues reduces alcohol intake via reduced affective associations rather than increased response inhibition. Addiction. 2012;107:1280-7.
2. Houben K, Nederkoorn C, Wiers RW, Jansen A. Resisting temptation: decreasing alcohol-related affect and drinking behavior by training response inhibition. Drug Alcohol Depend. 2011;116:132-6.
3. Petit G, Kornreich C, Noel X, Verbanck P, Campanella S. Alcohol-related context modulates performance of social drinkers in a visual Go/No-Go task: a preliminary assessment of event-related potentials. PLoS One. 2012;7:e37466.
4. Stein M, Fey W, Koenig T, Oehy J, Moggi F. Context-specific inhibition is related to craving in alcohol use disorders: a dangerous imbalance. Alcohol Clin Exp Res. 2018;42:69-80.
