# Supplementary material for: Learning to resist the urge: a double-blind, randomized controlled trial investigating alcohol-specific inhibition training in abstinent patients with alcohol use disorder
Source: Trials. 2019 Jul 5;20:402. doi: 10.1186/s13063-019-3505-2 (PMC6612135; doi:10.1186/s13063-019-3505-2)
Supplement: Supplementary file 5 — Overview of measures for healthy controls. (PDF 18 kb) [file 13063_2019_3505_MOESM5_ESM.pdf]

Additional File 5 Overview of measures for Healthy Controls

Healthy Controls		
	Construct	***Measure***
Screening	In-/Exclusion criteria	Clinical Information
	General psychopathology	BSCL
	Substance use	DUDIT
	Alcohol Use	AUDIT, AUD-S
Measurement	Demographics	Demographics
	Behavioral Inhibition - / Behavioral Approach System	BIS / BAS
	Impulsivity	I-8
	Sensation seeking	NISS
	Attention Deficit and Hyperactivity Disorder	ASRS-V1.1
	Antisocial Personality traits	ASPD
	Quality of life	WHOQOL-BREF
	Stress and Coping	SCI
	Alcohol expectancies	CAEQ
	Craving	OCDS-G
	Implicit association towards alcohol	IAT
	Inhibition (action restraint)	GNG
	Inhibition (action cancellation)	SST
(*only EEG group)*	Emotion & Emotion Regulation	Emo-Check
(*only EEG group)*	Alcohol use	HDL

Legend: The screening verifies in- and exclusion criteria, whereas the measurement qualifies the concepts of interest. The Emo-Check and HDL are only assessed in the EEG group. BSCL: Brief Symptom Check List; DUDIT: Drug Use Identification Test; AUDIT: Alcohol Use Disorders Identification Test; AUD-S: Alcohol Use Disorders Scale; BIS / BAS: Behavioral Inhibition System / Behavioral Approach System Scale; I-8: Scale for Impulsive Behavior; NISS: Need Inventory of Sensation Seeking; ASRS-V1.1: ADHD Self Report Scale; ASPD: Antisocial Personality Disorder; WHOQOL-BREF: WHO Quality of Life Scale; SCI: Stress and Coping Inventory; CAEQ: Comprehensive Alcohol Expectancy Questionnaire; OCDS-G: Obsessive Compulsive Drinking Scale; HDL: Health and Daily Living Form; IAT: Implicit Association Test; GNG: Go-NoGo Task; SST: Stop-Signal Task.
